# Biodegradable Elastomers and Gels for Elastic Electronics

**DOI:** 10.1002/advs.202105146

**Published:** 2022-02-25

**Authors:** Shuo Chen, Zekai Wu, Chengzhen Chu, Yufeng Ni, Rasoul Esmaeely Neisiany, Zhengwei You

**Affiliations:** ^1^ State Key Laboratory for Modification of Chemical Fibers and Polymer Materials College of Materials Science and Engineering Institute of Functional Materials Shanghai Engineering Research Center of Nano‐Biomaterials and Regenerative Medicine Institute of Functional Materials Donghua University Research Base of Textile Materials for Flexible Electronics and Biomedical Applications (China Textile Engineering Society) Shanghai 201620 P. R. China; ^2^ Department of Materials and Polymer Engineering Faculty of Engineering Hakim Sabzevari University Sabzevar 9617976487 Iran

**Keywords:** biodegradable, bio‐friendly, elastic electronics, elastomers, gels, implantable electronics, wearable electronics

## Abstract

Biodegradable electronics are considered as an important bio‐friendly solution for electronic waste (e‐waste) management, sustainable development, and emerging implantable devices. Elastic electronics with higher imitative mechanical characteristics of human tissues, have become crucial for human‐related applications. The convergence of biodegradability and elasticity has emerged a new paradigm of next‐generation electronics especially for wearable and implantable electronics. The corresponding biodegradable elastic materials are recognized as a key to drive this field toward the practical applications. The review first clarifies the relevant concepts including biodegradable and elastic electronics along with their general design principles. Subsequently, the crucial mechanisms of the degradation in polymeric materials are discussed in depth. The diverse types of biodegradable elastomers and gels for electronics are then summarized. Their molecular design, modification, processing, and device fabrication especially the structure–properties relationship as well as recent advanced are reviewed in detail. Finally, the current challenges and the future directions are proposed. The critical insights of biodegradability and elastic characteristics in the elastomers and gel allows them to be tailored and designed more effectively for electronic applications.

## Graphical abstract

Biodegradable elastic electronics are emerging as an important direction of next‐generation wearable and implantable electronics. Constructing elastic materials with the combination of unique biodegradability, mechanical, and electrical properties is the key for this field. The current development of biodegradable elastomers and gels used in electronics is summarized. The structure–property relationship is discussed. The future directions of this field are suggested.

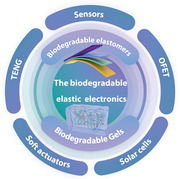

## 1 Introduction

The development of electronics has brought great convenience to human life and significantly improved the standard of healthcare, education, and security. There is an increasing dependence of human beings on electronics in daily life. Owing to the restriction of industrial materials, the consumer electronics have been extensively confined to rigid and bulky formats during the past decades. Recent rapid advances in wearable electronics have put favorable consideration of elastic electronic devices with soft, stretchable, resilient nature over traditional wafer electronics to better match the human tissues’ mechanical properties.^[^ ^1^, ^2^, ^3^, ^4^, ^5^ ^]^ Latest advances in electronic materials, process technologies, and integration design offer the exciting potential of elastic electronics for human‐machine interface,^[^ ^6^, ^7^, ^8^, ^9^ ^]^ soft robotics,^[^ ^10^, ^11^ ^]^ and implanted sensors.^[^ ^12^, ^13^, ^14^ ^]^ However, the rapid development of the electronics industry also brings a great challenge to e‐waste management.^[^ ^15^, ^16^ ^]^ The United Nations estimates that 50 million tons of e‐wastes are thrown away worldwide each year.^[^ ^17^ ^]^ Only a little of e‐wastes is recycled, while most of them are directly sent to landfills or incinerators. Thus, degradable electronic devices are highly desired to minimize the negative effects of e‐wastes on the environment.^[^ ^18^, ^19^ ^]^ In another hand, implantable electronics have become more and more important for healthcare. Most of them interact with soft tissues and are used in a mechanically dynamic environment, therefore, elastic electronics with biomimetic mechanical properties having resist cycling deformations are considered as ideal platforms.^[^ ^20^ ^]^ Furthermore, biodegradable electronics that can be safely absorbed by the body after completing their therapeutic and diagnostic functions are highly desired for various biomedical applications in this field.^[^ ^21^ ^]^ These biodegradable devices will avoid the need for a second surgical retrieval and reduce the probability of infection.^[^ ^22^ ^]^ Overall, the elasticity and degradability of electronic devices are becoming increasingly important for electronic devices, especially for wearables and implants.^[^ ^23^ ^]^

Nowadays, most costumer electronics immensely rely on various inorganic materials. The development of organic polymers with various desired properties provides a better choice for the preparation of bio‐friendly electronic devices. For elastic biodegradable electronics, the biodegradable elastomers and gels have been considered as the most promising candidate due to their soft tissue‐like mechanical properties, which enable conformal contact between wearable electronic devices and tissue's surface. However, progress in elastic biodegradable electronics has been largely hampered by the sluggish development of biodegradable elastic materials. The sheer volume of available choice of biodegradable elastic polymers is relatively insufficient for the various design and preparation of elastic electronics. The adjustable range of electrical properties (e.g., conductivity, mobility) and mechanical properties (e.g., stretchability, strength, toughness, conformability) cannot satisfy the requirement of present elastic electronics. To balance polymers’ biodegradability and other chemical, physical, and biological properties have been recognized as a unique challenge in the design and preparation of biodegradable elastic materials, which are required as follows.^[^ ^24^ ^]^ i) The processes and by‐products of elastic polymers degradation should not leave a permanent trace in environment. For implantable electronics, the degradation by‐products of elastic polymers should be able to be metabolized or absorbed by the body without persistent inflammatory or toxic reactions. ii) The biodegradation time of the elastic polymers should match the functional duration time of the elastic electronics. iii) The elastic polymers should have biomimetic mechanical properties and flexible processibility. The biodegradability of elastic polymers is vastly affected by their inherent properties, such as chemical structures, molecular weight, hydrophilicity, and degradation mechanism. Given the complexity of building biodegradable elastic polymers, it is necessary to summarize the rationally universal design concepts of biodegradable elastic polymers by figuring out the structure–function relationship between chemical structures and the manifested electrical, mechanical, and biodegradable properties.

**Figure 1:**
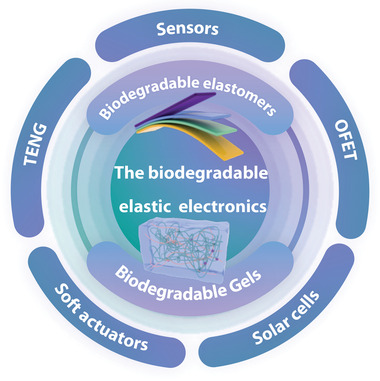
Schematic illustration of the most commonly used biodegradable elastic electronics based on elastomers and gels.

In this review, we discuss the molecular design, properties, and processing methods of developed biodegradable elastic polymers as well as their various applications of biodegradable elastic electronic devices (**Figure** **1**). Here, we also realized that some excellent reviews discussed biodegradable electronics.^[^ ^23^, ^25^, ^26^, ^27^ ^]^ Those reviews of biodegradable electronics mainly examine the organic plastic or inorganic materials without fully summarizing the recent discoveries in biodegradable elastic polymers. This article focuses on the molecular design of reported biodegradable elastic polymers, including elastomers and hydrogels, as well as the corresponding processing methods, providing analysis of advantages of these biodegradable elastomers over traditional materials used in electronics. This critical understanding provides the knowledge for the design of tailored materials and reveals the existing challenges for future research.

## 2 Overview

### 2.1 Biodegradable Electronics

According to different initiation methods and mechanisms, the degradation of polymers can be divided into chemical degradation, mechanical degradation, thermal degradation, and biodegradation.^[^ ^28^ ^]^ The chemical degradation relies on the active reagents in the environment, which could react with polymers to break down the molecular chains. Mechanical degradation is considered as the fracture of molecular chains induced by various mechanical forces (e.g., compression, shear, or tension forces). Thermal degradation refers to the decomposition of the polymers’ molecular structures in a high‐temperature environment. These three types of degradation generally involve harsh conditions or specific molecular structures. Different from the aforementioned degradation, biodegradation features an environmental‐ and human‐friendly strategy of degrading polymers, which has been shown great potential for green and sustainable electronic devices.

**Figure 2:**
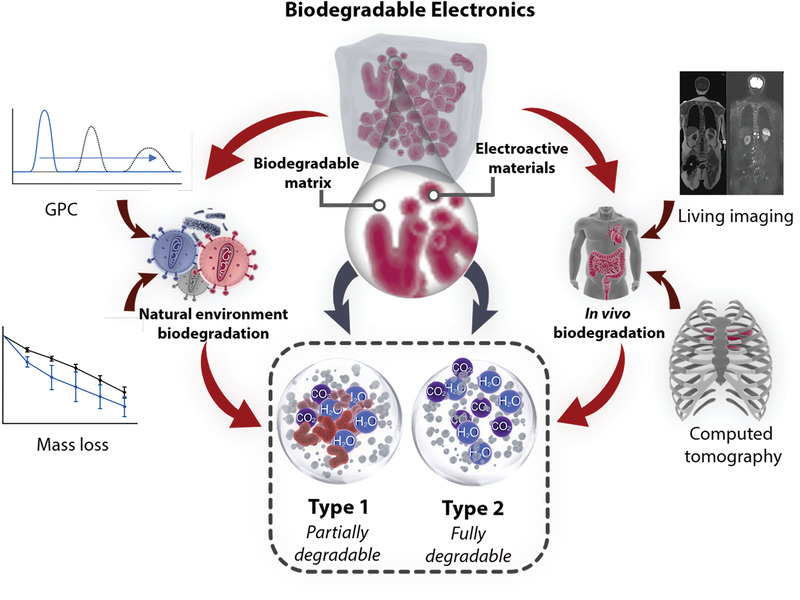
Schematic illustration of biodegradable electronics and their characterization methods.

The American Society for Testing and Materials (ASTM) regulated the term of “biodegradability” in ASTM D6400‐19 and ASTM D6868‐19, where a minimum of 90% of the plastic can be fully mineralized into the water, CO_2_, and biomass within 180 days under compositing conditions with the presence of fungi or microorganisms.^[^ ^29^ ^]^ This strict characteristic ensures the minimal detrimental effects of such plastics on the environment. It is important to note that since the biodegradable polymers used in biomedical applications have been recently developed, there is a lack of a clear standard to determine whether materials have promise for use as implanted electronics. For the biomaterials in medical use, Mikos and Temenoff defined the “biodegradation” as the “chemical breakdown of a material mediated by any component of the physiological environment (e.g., water, ions, proteins, cells, bacteria), into smaller constituents of low molecular weight products which are then processed, resorbed or cleared by the body.”^[^ ^16^ ^]^ It means that biodegradation of polymers involves chemical or biological breakdown into smaller fragments that could be dissolved or metabolized, which happens in biologically benign or physiological conditions.^[^ ^30^ ^]^ **Figure** **2** schematic shows the biodegradable electronics and their characterization methods. The typical biodegradable electronics with desired electronic properties are generally composed of electroactive material (red) and biodegradable matrix (gray) through hydrolysis or oxidation by enzymes.^[^ ^31^ ^]^ For normal degradation tests, there are two different degradation conditions are commonly used: natural environment and in vivo. Different characterization methods are used according to the degradation conditions. Depending on the molecular structures and composition of used materials, the biodegradable electronics would be divided into two types 1 (partially degradable) and 2 (fully degradable). The polymers in the environment are degraded mainly by the metabolic activity of microbial communities. However, whatever the degradation conditions and mechanisms, the key point is the cleavable chemical linkages in the molecular chains of polymers. The common groups that are susceptible to hydrolysis generally contain C═O bonds linked to other heteroelements (oxygen, nitrogen, sulfur) and provide cleavage sites of the molecular backbone in biologically benign environments.^[^ ^30^ ^]^ Biodegradation of polymers is a complex and systematic process, which is affected by many factors. The inherent properties of the polymers, such as molecular weight, hydrophilicity, porosity, and crystallinity, would influence the degradation rate by determining the water‐absorbing capacity. These parameters can be engineered during the synthesis of new polymeric materials for electronic application in order to tailor the biodegradation of electronic device. Beyond that, the ions, enzymes, and microorganisms of different degradation conditions also exhibit a tremendous impact on biodegradation rates and mechanisms.^[^ ^26^ ^]^

The characterization methods performed to evaluate the degradability of polymers vastly depend on the specific biodegradation conditions and mechanism.^[^ ^29^ ^]^ For the in vitro experiments, the mass loss profiles of polymers are the most commonly used method to characterize the biodegradation of polymers. Besides, other characterization methods, including gel permeation chromatography, Fourier transform infrared spectroscopy, and scanning electron microscopy (SEM) could precisely present the change of chemical structures and morphologies of polymers during the biodegradation process. Some reports also introduced nuclear magnetic resonance and liquid chromatograph‐mass spectrometer to further confirm the biodegradation in the structure of products.

The in vivo investigation of the polymer biodegradability was much complex compared with in vitro evaluations. The experimental animals were killed at each specific time point and the material was taken out for weighing, thus the degradation curve of the material reflected by the obtained mass changes. However, there are some shortcomings for this method. First, the taking out procedure of implanted materials needs to kill the animals, which potentially breaks the continuity of polymers’ degradation experiments. Second, to avoid large deviation of experimental data due to the individual specificity of experimental animals, there are large size groups of animals for the experiments. Third, the disintegrated polymers could easily adhere to surrounding tissues during the degradation process. It is very difficult and time‐consuming to isolate the implant material, further resulting in the inaccuracy of degradation data. The monitoring of the morphology changes of the polymers during the degradation process, while keeping the animal alive is developed characterization methods in decades. Noninvasive and real‐time living molecular imaging has become an important method for in vitro degradation data collection. Commonly used technologies include magnetic resonance imaging, computed tomography, ultrasound imaging, bioluminescence imaging, fluorescence imaging systems.^[^ ^32^, ^33^, ^34^, ^35^, ^36^ ^]^ Their ability to capture multiple images vertically provides reliable information of implanted materials’ degradation while reducing the number of experimental animals. Meanwhile, three dimensional (3D) image advanced processing, mechanical analysis, and other related analysis can be performed by software. Whether on research or in clinical applications, in vivo imaging and analysis have gained great progress with the developments of biological techniques, which demonstrated a promising development prospect.

The ideal biodegradable elastic electronic devices would be able to function within a designed timeframe before breaking down into harmless components and disappearing.^[^ ^37^ ^]^ Since most biodegradable elastomers are naturally insulating, it is still challenging to fabricate fully biodegradable electronics, especially semiconductors and electrodes. Bao and co‐workers put forward a new standpoint on the degradation of electronics, which can be classified into two categories: disintegrable, partially degradable (named Type 1) devices and fully degradable (Type 2) devices.^[^ ^26^ ^]^ Only the matrix of Type I devices is degradable, which composite nondegradable electrical active materials. The devices can be broken down into small molecule building blocks at last. For Type 2 devices, all the components can be fully broken down into monomers or oligomers. Incorporating the electrical active moieties into the biodegradable polymer backbones could enable fully biocompatible conductors or semiconductors, which makes Type 2 devices available. However, due to the complex chemical synthesis and devices integration technologies, fewer works about Type 2 devices have been reported so far. No matter which degradation mechanisms, ensuring the safety and biocompatibility of degradation by‐products is the most important task when deploying the electronic devices within an ecosystem.

### 2.2 The Basic Chemistries of Biodegradation

#### 2.2.1 Hydrolytic Degradation

**Figure 3:**
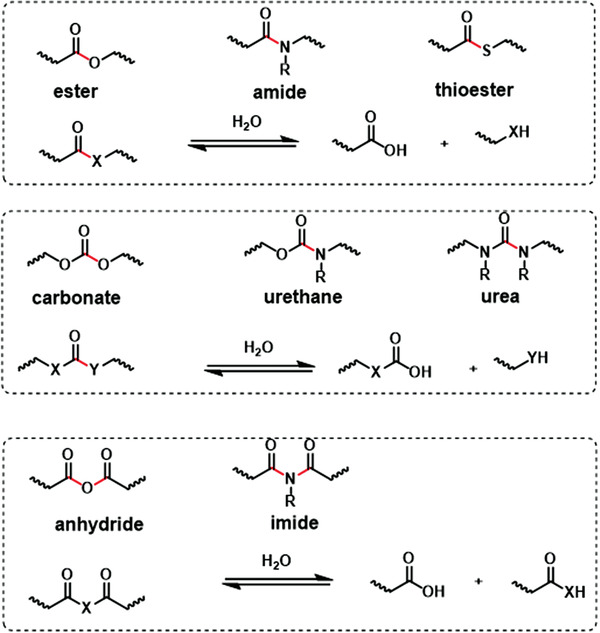
The hydrolyzable groups containing C═O bonds and the possible chemistry reactions. The hydrolyzable bonds are marked with red color. Reproduced with permission.^[^ ^30^ ^]^ Copyright 2008, Elsevier.

Heterochain polymers, especially those containing oxygen and/or nitrogen atoms in the backbone are more susceptible to be hydrolyzed.^[^ ^30^ ^]^ The cleavage of these labile bonds is induced by water and catalyzed by bases, acids, or enzymes. As shown in **Figure** **3**, the groups that are preferable to hydrolysis usually contain esters, thioesters, carbonates, urethanes, anhydrides, amides, etc. The similarity of these functional groups is generally containing C═O bonds linked to other heteroelements (oxygen, nitrogen, sulfur), which provide cleavage sites on the molecular backbone for degradation. Another category of the functional groups, such as ethers, sulfonates, polyphosphonates, and cyanoacrylates are also readily hydrolyzed by a catalyzed acid or base.

The chemical structures and amount of hydrolyzable groups dominate the hydrolysis rate of materials. Besides, the polymer characteristics including hydrophilicity, surface area, and crystallinity also influence the hydrolysis rate by determining the rate of water uptake. Generally, the materials with high hydrophilicity, low crystallinity, less‐crosslinked structure, and high surface area (porous materials or materials with a rough surface) tend to possess a high hydrolysis rate which can be controlled during the synthesis of new materials for electronic applications.

**Figure 4:**
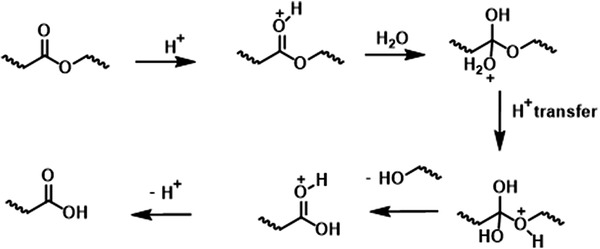
The mechanism for acid‐catalyzed ester bonds hydrolysis, which is cleaved to yield carboxylic acid and alcohol. Reproduced with permission.^[^ ^30^ ^]^ Copyright 2008, Elsevier.

Except for the intrinsic properties, the surrounding environments of materials also greatly influence hydrolysis rate.^[^ ^38^ ^]^ The biological and natural environments typically contain ions, such as H^+^, Na^+^, K^+^, Mg^2+^, Ca^2+^, OH^−^, HCO_3_^−^, Cl^−^, H_2_PO_4_^2−^, and SO_4_^2−^ could catalyze the hydrolysis rate, which depends on the salt diffusion into polymers determined by the hydrophilicity of the materials. For example, the hydrolysis of ester bonds may be catalyzed considerably by the pH of salt in the environment (**Figure** **4**). Enzymes generated by the cells or microorganisms can greatly promote hydrolytic degradation both in vivo and in vitro.^[^ ^31^ ^]^ Since the large size of enzyme molecules, they are unable to deeply penetrate into materials, thereby they usually induce surface degradation. Utilizing hydrolyzable bonds as linkages to construct electronic biodegradable materials is so far the most common method. Based on comprehensive consideration of factors, the degradation time of the materials can be readily adjusted within a range of a few days to several years for specific applications.

#### 2.2.2 Oxidative Degradation

**Figure 5:**
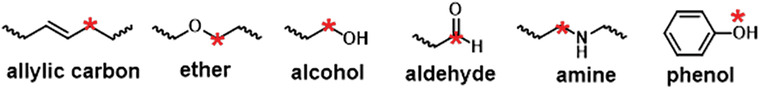
Chemical structures of moieties susceptible to oxidative. The red stars represent the susceptible points. Reproduced with permission.^[^ ^30^ ^]^ Copyright 2008, Elsevier.

Oxidation degradation is also another biologically relevant process. The free radicals generated chemically or enzymatically by living organisms mainly induce the degradation of the materials. As shown in **Figure** **5**, the functional groups containing the carbon substituted by allylic, ethers, phenols, alcohols, aldehydes, and amines group, are the most possible oxidative degradation.^[^ ^30^ ^]^ Unlike the hydrolysis degradation process, the oxidative degradation generally contains three stages: initiation, propagation, and termination.^[^ ^39^ ^]^ The absorbed energy from an external source would trigger the initiation reaction, by causing the cleavage of the covalent bonds. After the initiation reaction starts, the propagation may take place by unzipping. The cleavage of covalent bonds would generate radicals, which could be transferred to another chain or abstract the neighboring hydrogen atoms to generate new radicals in the same chain.

The triggered energy for oxidative degradation of the polymers ranges from 30 to 90 kcal mol^−1^.^[^ ^39^ ^]^ Thus, the reactions usually need an external energy source, such as heat, light, or radiation. For the oxidative degradation that happens in vivo, the process is triggered by the immune response in the body. The activated phagocytes, such as polymorphonuclear neutrophils and macrophages, swallow and try to digest the foreign materials. Studies have found that the macrophages and neutrophils can release reactive oxygen species around the implanted materials during the phagocytosis process,^[^ ^40^ ^]^ which is caused by the one‐electron reduction of oxygen to superoxide anion catalyzed by the nicotinamide adenine dinucleotide phosphate (NADPH) oxidase and using NADPH as substrate. Specific enzymes (e.g., peroxisome) are capable to transfer the superoxide anion to hydrogen peroxide (H_2_O_2_) by the superoxide dismutase. These substances are relatively harmless on their own but can be converted to hydroxyl radicals in the presence of iron or other transition metal catalysts and trigger oxidation on the polymer surface. In addition, the oxidant hydroxyl radicals also play an important role in elimination of invading microorganisms by the phagocytes. Hydrogen peroxide is converted to hypochlorous acid (HOCl) by myeloperoxidase produced by lysosomes in neutrophils. The HOCl could oxidize the nitrogen functional groups (amide, urethane) and possibly break these bonds.^[^ ^41^ ^]^

### 2.3 Elastic Electronics

Currently developed flexible and stretchable forms of electronics open up the next‐generation wearable and implanted electronic applications.^[^ ^42^, ^43^, ^44^ ^]^ These devices could mimic the mechanical properties of biological soft tissues, which result in conformal and seamless contact to irregular and moving surfaces of human bodies. For the implanted applications, the soft nature of flexible and stretchable devices could reduce the possibility of inflammatory responses caused by the stiff implanted devices.^[^ ^45^ ^]^ The flexible electronics are one of the earliest concepts of soft electronics.^[^ ^46^, ^47^ ^]^ The initial pursuit of the flexible electronics is that these devices could maintain their functions under bending, twisting, and even slight stretching states. With the development of elastic electronics materials and integrated technologies, researchers further realized maintaining of electrical functions of electronics under tensile large strains, which was defined as stretchable electronics.^[^ ^48^, ^49^ ^]^ However, the actual applications of these soft wearable and implantable electronics are biological environments generally involving reversible and dynamic deformations. Though a significant portion of the stretchable electronics has already achieved elastic properties, the term “flexible” or “stretchable” cannot comprehensively reflect the requirement and further development of soft wearable and implantable electronics. Thus, we come up with the concept of “elastic electronics” for purpose of accurately reflecting the future development of soft electronics. This new term extends the concepts of flexible and stretchable electronics by emphasizing the resilience of the devices. The elastic electronic devices should recover their original state after the removal of external force in a relatively short time and could sustain reversible deformation while keeping their electrical properties.^[^ ^50^ ^]^ The stability and repeatability of electrical signals under dynamic environments are the most important characters of elastic electronic devices. Considering the environmental‐ and human‐friendly perspective, constructing elastic electronics with biodegradability would have great potential in healthcare, energy, and artificial intelligence purposes. Therefore, biodegradable elastic electronics is an emerging concept of wearable and implanted electronics that possess a biodegradable behavior as well as biomimetic mechanical properties, which could precisely define the development direction of next‐generation wearable and implantable electronics.

**Figure 6:**
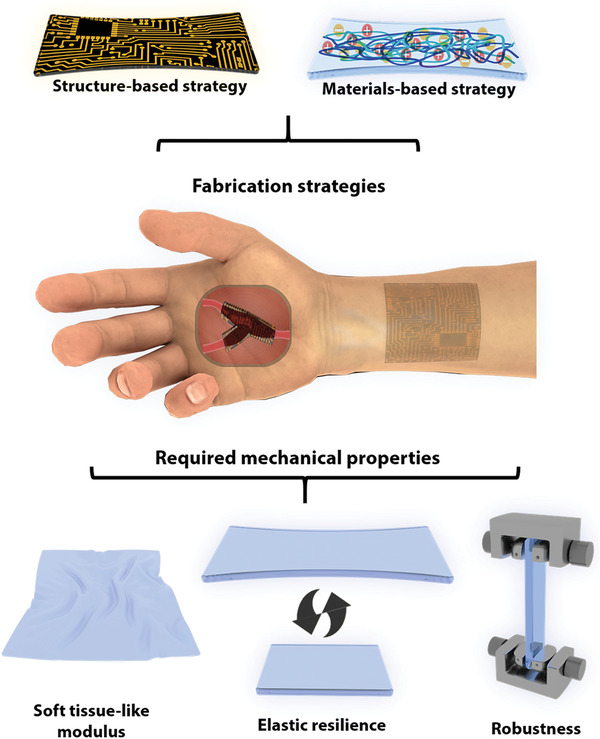
Schematic illustration of elastic electronics, exhibiting their common fabrication strategies and required mechanical properties.

Recently developed fabrication methods of biodegradable elastic electronics involve the innovation of material synthesis, mechanical structural design, and integration strategies. To date, there are two established strategies to achieve the elasticity of electronic devices: structure‐based and material‐based strategies (**Figure** **6**).^[^ ^51^ ^]^ The first strategy originates from traditional electronic device processing and involves constructing patterns (e.g., fractal and serpentine) of nonstretchable conductive materials on the substrates.^[^ ^52^ ^]^ The buckling structures of these patterns could enable the devices to tolerate the applied strains and perform their functions appropriately. Although these structural strategies enhance strain tolerance, the inherent rigidity of traditional electronic materials may, unfortunately, lead to mechanical mismatches between wearable or implantable devices and human soft tissue.^[^ ^51^ ^]^ Another approach to elastic electronic systems directly involves the preparation and processing technologies of intrinsically stretchable materials to endow the intrinsic elasticity to individual device components and their integrated systems. Elastomeric polymers, including elastomers and gels, with essentially viscoelastic deformation behavior, would be a superior platform to be compositing and patterning for the preparation of elastic electronics.^[^ ^53^, ^54^ ^]^ Various inorganic nanomaterials and conjugated polymers have been widely used as electronic fillers to composite with biodegradable elastic polymers.^[^ ^51^ ^]^ These fillers form percolation networks enabling continuous carrier movement even under stretched, which makes the combination of conductivity and elasticity. The polymers could be processed by common processing technologies, such as molding, spinning, 3D printing, and micro–nano fabrication to successfully fabricate elastic integrated electronic devices.

As a newly developed field, a clear understanding of desirable properties can effectively promote the further advancement of biodegradable elastic electronics. The primary task of establishing biodegradable elastic electronics is to achieve the combination of elasticity and biodegradability.^[^ ^2^, ^55^, ^56^ ^]^ For mechanical properties, Young's modulus of the electronics materials are the key properties.^[^ ^57^ ^]^ The materials with low modulus can effectively improve wearing comfort and superior electrical performance by enabling intimate and conformal contact with curvilinear human soft tissue with dynamic deformation.^[^ ^1^ ^]^ Thus, the wearable and implantable electronic devices (especially e‐skins) must be soft enough to be capable of deforming to conform to the topography of the tissues. Another important mechanical property of materials is stretchability. In daily activities, human bodies are always in continuous movement with reversible stretchability up to 100% of skins.^[^ ^58^ ^]^ It is primarily to construct elastic electronics by introducing materials with relatively high stretchability to accommodate the strain during natural body motion as well as the induced complex topography. For instance, compositing nanostructured conductive materials as fillers with soft and stretchable substrates can endow the devices with flexible mechanical performance, by which that they can bend, wrinkle, or stretch. Beyond that, mechanical resilience is the important property of the materials, by which the “elastic” electronics distinguish them from the “stretchable” electronics.^[^ ^59^ ^]^ The resilience of materials enables the electronic devices to recover to the original state upon the external force is removed. For the biological tissues or organs with reversible deformation (e.g., heart, joint, blood vessel), the electronic devices with excellent resilience can better fit their morphology and adapt to their movement, which is beneficial for the accurate sensing and efficient energy harvesting. The monitoring of high‐frequency body motions induced by energetic exercise for athletes or soldiers is always a challenging task, electronic devices with excellent resilience would be an ideal candidate, which can be capable of tolerating high‐frequency motion. Besides, the robustness of materials is also important for the long‐term stability of elastic electronic devices.^[^ ^60^ ^]^ Within the service life of electronic devices, the devices will be subjected to tens of thousands of reversible deformations including bending, twisting, and stretching. To ensure the stability and repeatability of electrical signals, electronic materials with excellent robustness are desirable.

## 3 Biodegradable Elastic Materials for Electronics

The fundamental components of the common electronic prototype can be simplified into the substrate, dielectric, semiconductors, electrodes, and encapsulants.^[^ ^26^ ^]^ Since most biodegradable elastomers are naturally insulating, a large number of biodegradable and stretchable materials have been developed for use as substrates, dielectric, and encapsulants. These three components take up a major proportion of electronic devices’ mass, which determines the overall elastic and biodegradable performance of the devices. Besides, compositing biodegradable materials with inorganic nanomaterials, polymeric conductors, and semiconductors or copolymerizing the oligomers of polymeric conductors and semiconductors with biodegradable elastomers molecular backbones have been demonstrated as effective strategies to endow biodegradable elastomers with electrical properties.^[^ ^61^, ^62^ ^]^ The as‐prepared biodegradable conductors and semiconductors could serve as electrical active components to achieve the overall elastic mechanical design of electronic devices. As for the gels, such as hydrogel, their soft nature and intrinsic ionic conductivity enable them outstanding candidates for the elastic substrates and conductors.^[^ ^63^, ^64^ ^]^ Compositing with inorganic nanomaterials and conducting polymers are also widely used to endow gels with decent conductivity. In this section, we provide an overview of the chemistries, molecular designs, and electronic applications of biodegradable elastic materials.

Recently some elastomers including polyglycerol sebacate (PGS), PGS derivatives, poly(1,8‐octanediol‐*co*‐citrate) (POC), urethane‐based elastomers, and dynamically covalent cross‐linked elastomers have been developed for electronic applications. Since the chemical structure of these materials plays a crucial role in their properties and performance, the latest achievements are reviewed in the following subsections and the results are summarized in **Table** **1**.

**Table 1:** Summary of recent advances in the development of elastomers for electronic applications

Materials	Electrical property	Potential component in devices	Remarkable results
PGS	Insulating	Substrates, dielectric, and encapsulants	Widely used electronic elastomers with moderate modulus (0.03 to 1.2 MPa) and biodegradable rate (17.6% after 60 days in vitro and 100% after 100 days in vivo).^[^ ^70^ ^]^
PGSA	Insulating	Substrates, dielectric, and encapsulants	Endowing the photo‐crosslinking properties with PGS elastomers by grafting the acrylate groups to the backbone of PGS.^[^ ^91^ ^]^
PSeD‐U	Insulating	Substrates and dielectric	Constructing hybrid cross‐linking structures to improve the strength and toughness while keeping the low modulus of PGS elastomers.^[^ ^106^ ^]^
POC	Insulating	Substrates, dielectric, and encapsulants	Widely used polyester elastomers with mild curing conditions and a complete degradation after 180 days when soaking in PBS at 37 °C.^[^ ^108^ ^]^
PSeHCD	Insulating	Substrates, dielectric, and encapsulants	Unique alternating polyester‐co‐polyurethane structure endowing the elastomers with excellent biodegradability and excellent self‐healing properties at room temperature.^[^ ^116^ ^]^
PFB	Insulating	Substrates, dielectric, and encapsulants	Dynamic covalently cross‐linked elastomers with a combination of biodegradability, processability, and recyclability.^[^ ^120^ ^]^
PGSAP	Conductive (doped)	Electrodes	Biodegradable elastomers with an intrinsic conductivity up to 8.5 × 10^−5^ S cm^−1^ after doped with camphorsulfonic acid.^[^ ^62^ ^]^
DCPU	Conductive	Electrodes	Dopant‐free conductive and biodegradable elastomers with a unique strain‐induced increase of conductivity.^[^ ^124^ ^]^
E‐PCL/p(DPP–PPD)	Semiconductive	Semiconductors	Fully degradable and semiconductive elastomers with stable mobility (≈0.03 cm^2^ V^−1^ s^−1^) at strains up to 100%.^[^ ^61^ ^]^

### 3.1 Biodegradable Insulting Elastomers

#### 3.1.1 Polyester Elastomers: PGS

Polyesters are inherently biodegradable owing to the ability of ester bonds can be broken down by hydrolysis or enzymatically in the body, or both.^[^ ^65^, ^66^ ^]^ Among the various polyester elastomers, the ones synthesized by thermal polycondensation of polyol and polycarboxylic acid are the most representative category.^[^ ^24^, ^67^, ^68^ ^]^ PGS is one of the most well‐known polyester elastomers, which have been widely used in the biomedical fields of tissue engineering, drug delivery, and biological adhesives.^[^ ^69^, ^70^ ^]^ The raw materials glycerol and sebacic acid are endogenous metabolites of the human body and have been widely used in the food, biomedical, and cosmetic industries. In addition, no catalysts or additives are used in the PGS polymerization process, eliminating the potential toxicity of application in vivo. PGS elastomers are typically prepared in two steps: the first step is the synthesis of prepolymer via the polycondensation of glycerol and sebacic acid, the second step is cross‐linking. The resultant PGS elastomers are transparent, almost colorless with high elasticity, and tuned biodegradation. PGS elastomers are designed to mimic the mechanical properties of elastin by constructing 3D cross‐linked networks in the material. PGS has been reported to be completely amorphous above 37 °C.^[^ ^71^ ^]^ Thus, PGS elastomers exhibit typical nonlinear stress–strain mechanical behavior similar to that of vulcanized rubber with Young's modulus ranging from 0.03 to 1.2 MPa, the tensile strength nearly 0.5 MPa, and elongation at break of more than 300%.^[^ ^71^ ^]^ The elastic mechanical properties of PGS elastomers originate from the covalent cross‐linking of the network and hydrogen bonding interactions of hydroxyl groups between random coils. The mechanical properties of PGS elastomers can be readily tuned by the feeding ratio of monomers as well as the curing time and temperature.^[^ ^72^ ^]^

In addition to elastic mechanical properties, the biodegradability of PGS elastomers is another merit for its attractiveness in biomedical applications. Owing to the hydrolyzable and enzymatic degradation of ester bonds, the PGS elastomers exhibit considerable degradability both in the natural environment and in vivo.^[^ ^73^, ^74^, ^75^ ^]^ PGS elastomers have been demonstrated in vivo degradation behavior of surface erosion of enzymatic digestion, which allows the linear mass loss with degradation time, good preservation of geometries, and mechanical strength. However, most biodegradable polyester plastics, such as polylactic acid (PLA), polycaprolactone (PCL), and polylactic acid–glycolic acid (PLGA) perform the mechanism of bulk degradation, which might result in severe geometric collapse and unpredictable loss in integrity during the degradation process.^[^ ^69^ ^]^ It is worthy to note the unique surface erosion mechanism enables the good retention of performance of PGS elastomers during the degradation process, which is beneficial to the stable electrical functionality of the implantable electronic devices. Studies have found that the degradation rate of PGS elastomers in vivo was much faster than in vitro (phosphate buffer solutions) (17.6% after 60 days in vitro vs 100% after 100 days in vivo).^[^ ^70^ ^]^ The biodegradation rate of PGS elastomers is closely related to the degree of cross‐linking. Results showed that in vitro degradation rate of PGS elastomers was 0.6–0.9 mm per month after 2 days of cross‐linking, while the degradation rate of PGS elastomers after 7 days of cross‐linking was 0.2–0.6 mm per month.^[^ ^75^ ^]^ These results indicated that adjusting the curing time or temperature could tune the degradation rate of PGS elastomers.^[^ ^76^ ^]^ As a well‐known biomaterial, PGS elastomers have shown excellent biocompatibility. The degradation products of PGS are glycerol and sebacic acid, which are the body's natural metabolites. Various studies of in vivo and in vitro biocompatibility tests results have demonstrated that PGS elastomers are an excellent candidate for implantable applications.^[^ ^77^, ^78^, ^79^, ^80^ ^]^

**Figure 7:**
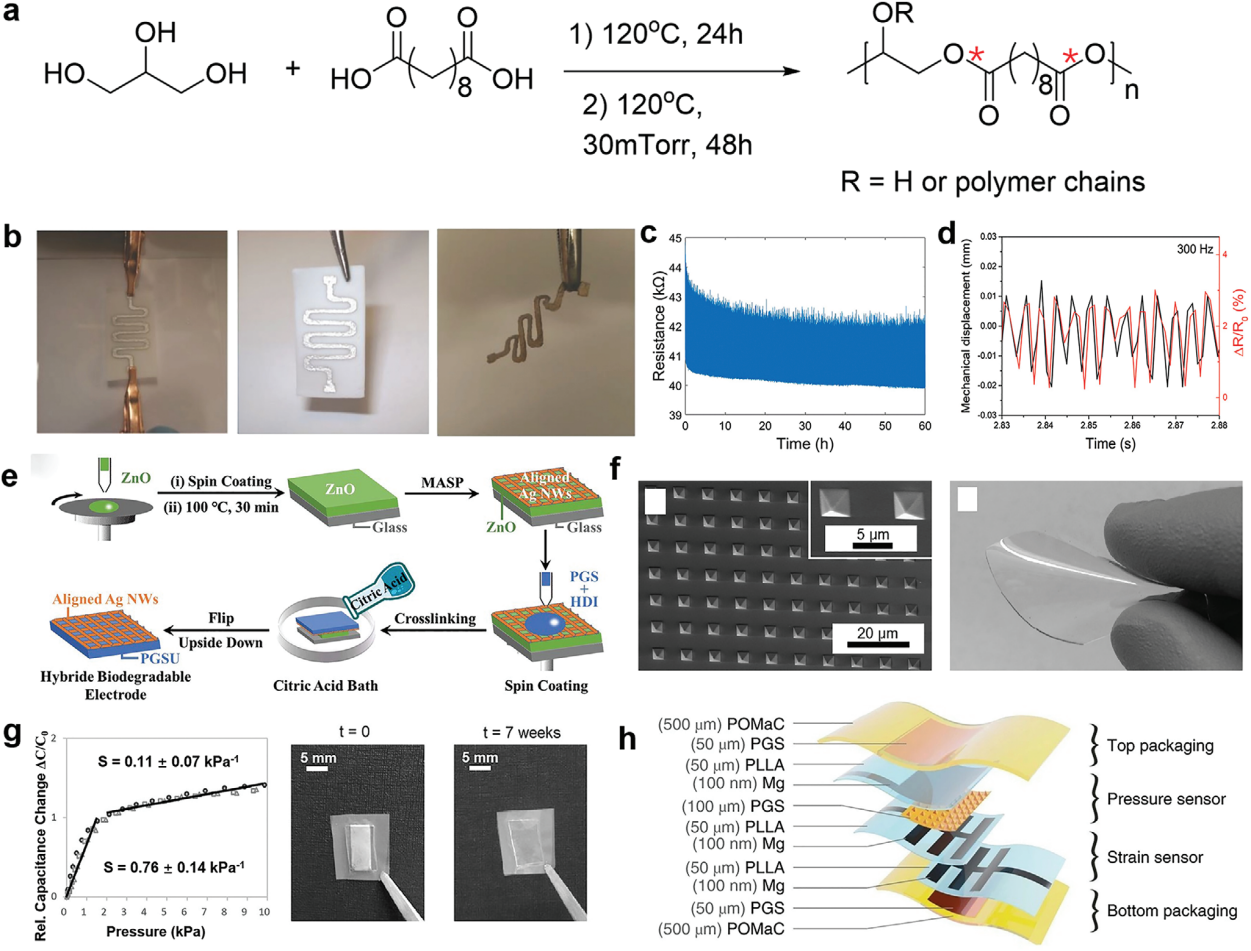
The chemical structure and application of PGS elastomers in electronics. a) Chemical structure of PGS, with emphasis on the susceptible sites (red stars) for biodegradability. b) The degradation of the device made from PGS, inside a solution of 0.5 m NaOH. Reproduced with permission.^[^ ^81^ ^]^ Copyright 2014, Wiley‐VCH. c) Evolution of the piezoresistive response after 1 200 000 cycles. d) Electromechanical shaker displacement and the change of resistance reported at 300 Hz. Reproduced with permission.^[^ ^82^ ^]^ Copyright 2019, Wiley‐VCH. e) Schematic illustration of fabrication procedure for flexible, hybrid, and biodegradable electrodes containing cross‐aligned Ag nanowires (Ag NWs) impregnated in PGS film. Reproduced with permission.^[^ ^85^ ^]^ Copyright 2020, Wiley‐VCH. f) SEM images of the microstructured PGS films(left). The image of PGS biodegradable elastomer film (right). g) The average sensitivity and standard deviation over all the 20 sensors with an array of 4 × 5 elements. Reproduced with permission.^[^ ^86^ ^]^ Copyright 2015, Wiley‐VCH. h) Materials and overall assembly of the fully biodegradable strain and pressure sensor. Reproduced with permission.^[^ ^87^ ^]^ Copyright 2018, Springer Nature.

As a kind of natural insulating elastomers, PGS elastomers are widely used as substrate and dielectric materials in biodegradable elastic electronics. Najafabadi et al. prepared elastic sheets of poly(caprolactone)–poly(glycerol sebacate) (PGS–PCL) by electrospinning as substrates for engineering elastic and biodegradable electronics (**Figure** **7**a).^[^ ^81^ ^]^ Different conductive patterns based on silver ink were created on the surfaces of the PGS–PCL substrates by screen printing through a shadow mask (stencil). The temperature sensors, heaters, and strain sensors were successfully fabricated on PGS–PCL substrates. The elastic moduli of the substrate and the patterned PGS–PCL sheets were 4.86 ± 0.54 and 4.87 ± 0.17 MPa, respectively. During the cyclic tensile test, the stress–strain curves showed slight variation after the third cycle. However, the electrical resistance did not change during the first few cycles and increased significantly after 20 cycles, which may be ascribed to the morphology changes of the PGS–PCL sheets during reversible deformations. The electronic devices were observed a mass loss of 15% and 20% when incubated in phosphate buffer saline (PBS) and NaOH (0.01 m) solution after 14 days, respectively. During the degradation process, the architecture and electrical functionality of the fabricated systems were well preserved. Sencadas et al. reported a resistive‐type sensor based on the PGS elastomers with foam‐like structures and electromechanical properties. The multiwalled carbon nanotubes (MWCNTs) were used as conductive fillers composited with PGS prepolymers with the salt particles as porogen.^[^ ^82^ ^]^ After the thermal cross‐linking process, the salt particles were dissolved by water, resulting in porous foam‐like structures. The PGS/MWCNTs foams presented an elastic modulus of 37.9 ± 0.9 kPa with 7 wt% conductive filler. As shown in Figure 7b, the foam‐like sensors showed a remarkable elastic behavior with little mechanical damping, resulting in excellent repeatability, and reliability with a long lifetime (>1 200 000 cycles). Besides, the resultant sensors exhibited high sensitivity (GF of −9.1 ± 0.9) and fast response (≤3 ms with a bandwidth well above 300 Hz) indicating the great potential as pressure sensors (Figure 7c). The graphene and poly(3,4‐ethylene dioxythiophene):poly(styrene sulfonate) (PEDOT:PSS) were also used as conductive components and composited with PGS elastomers to fabricate biodegradable elastic electronic devices.^[^ ^83^, ^84^ ^]^

Qi et al. reported a strategy for judiciously constructing flexible perovskite solar cells (PSCs) based on the PGS elastic substrates.^[^ ^85^ ^]^ Aliphatic hexamethylene diisocyanate was used as the cross‐linking agent to improve the mechanical strength. Crossly aligned silver nanowires (Ag NWs) by meniscus‐assisted solution printed were transfer printed to the PGS elastic substrates to obtain elastic hybrid electrodes with enhanced electrical conductivity (52.6 Ω per square) at 90% transparency (Figure 7d). After depositing perovskite film onto the hybrid biodegradable electrode, the resulting flexible PSCs manifested excellent optoelectronic properties with a power conversion efficiency of 17.51% as well as appealing mechanical robustness against bending. The PSCs carry a 93.2% retention of initial power conversion efficiency at a bending radius of 9.3 mm after 500 bending cycles, demonstrating their superior mechanical flexibility. Owing to the excellent biodegradability of PGS substrate, the PSCs can be easily biodegraded in the lipase water solution, leaving high valued Ag NWs that could be recycled.

Except for being used as a substrate, PGS elastomers are also promising candidates as dielectric materials due to their intrinsic insulating properties. Boutry et al. prepared a capacitive pressure sensor with a sandwich structure.^[^ ^86^ ^]^ The thin films of PGS elastomers acted as elastic dielectric layers, while the magnesium and iron served as electrode materials. For better electrical performance, the “pyramid” micrometer‐scale structures were fabricated on the surface of the PGS dielectric using the template method (Figure 7e). The pressure sensor exhibited a sensitivity of 0.76 ± 0.14 kPa^−1^ in low pressure regime (*p* < 2 kPa) and 0.11 ± 0.07 kPa^−1^ at higher pressures (2 < *p* < 10 kPa) (Figure 7f). After applying a constant pressure of 80 kPa and releasing the force for more than 8000 cycles, the variation of pressure sensitivity was reported less than 10%, indicating excellent elastic resilience. The pressure sensor could feel a rice grain with a weight of 5 mg and detect heart rate differences in different parts of the body. Due to the good biodegradability of PGS elastomers and magnesium and iron metals, the device was fully biodegradable after use, with a degradation rate of about 0.2–1.5 mm per month in vivo. This type of biodegradation was suggested to avoid secondary surgical injury during removal and reduce electronic contamination. Based on this, Boutry et al. subsequently developed a thin‐film implantable sensor based on wireless signal transmission with potential to be used for local blood flow monitoring after complex vascular suture reconstruction surgery (such as cardiovascular surgery, transplantation surgery, etc.).^[^ ^87^ ^]^ The main component of the device was a pressure capacitance sensor with PGS elastomer as dielectric material (Figure 7g). The developed part was able to surround the blood vessel and convert the degree of blood vessel expansion into a capacitance signal. With the oscillating circuit made of magnesium metal and PLGA biomaterials, the change of capacitance led to the shift of resonant frequency in the circuit, which was monitored through the skin wirelessly via inductive coupling, thus achieving wireless, passive in vivo sensing. The device showed millisecond response time and stable cycle performance as well. The practical application of the device was demonstrated in vitro using a custom‐built artificial artery model, and subsequently demonstrated in animal experiments in rats.

#### 3.1.2 Polyester Elastomers: PGS Derivatives

**Figure 8:**
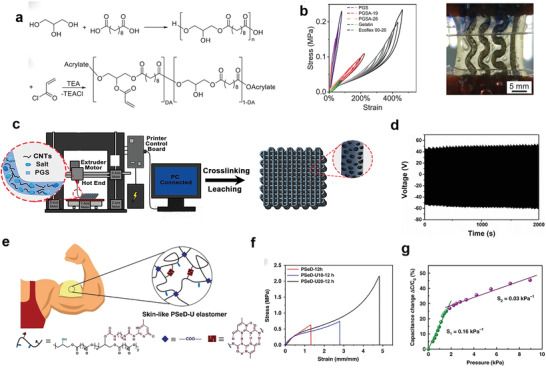
The modification and 3D printing of PGS elastomers for electronic applications. a). Synthesis scheme of PGSA from PGS. b) Stress–strain curves for biodegradable substrate candidates and an Ecoflex reference in cyclic linear tensile test (left). Embedded serpentine Galinstan interconnects in gelatin and PGSA‐19 for cyclic tensile tests (right). Reproduced with permission.^[^ ^91^ ^]^ Copyright 2021, Wiley‐VCH. c) Schematic diagram of the fabrication of the 3D printing TENGs (3DP‐TENGs) and the hierarchical porous structure. d) The durability test of 3DP‐TENGs. Reproduced with permission.^[^ ^92^ ^]^ Copyright 2018, Wiley‐VCH. e) The schematic illustration of the skin‐like PSeD‐U elastomers with physical and covalent hybrid cross‐linked structures. f) Typical tensile stress–strain curves of PSeD‐U‐12h elastomers with different densities of hydrogen bond while practically identical covalent cross‐linking densities. g) Pressure–response curve of the piezocapacitive pressure sensor. Reproduced with permission.^[^ ^106^ ^]^ Copyright 2020, Springer Nature.

Though PGS elastomers exhibited promising prospects in biodegradable elastic electronics, the inherent poor processability limited their further applications.^[^ ^76^, ^88^, ^89^, ^90^ ^]^ However, as thermoset polymers, PGS elastomers are difficult to process once the cross‐linking procedure is completed. Besides, the cross‐linking procedure requires harsh conditions of high temperature (high than 100 °C) and vacuum (10 mTorr) as well as long reaction time (several days), which vastly limit the scalable preparation of PGS elastomers. To overcome these shortcomings, new processing strategies were emerged based on photo‐crosslinking and 3D printing. Held et al. synthesized photo‐crosslinkable elastomer poly(glycerol sebacate) acrylate (PGSA) and demonstrated its applicability in soft and stretchable electronics (**Figure** **8**a).^[^ ^91^ ^]^ The obtained PGSA‐19 (19% degree of acrylation) elastomers showed a Young's modulus of 80 kPa, the high ultimate tensile strength of 265 kPa, and the high elongation at break of 350%. The small hysteresis during cyclic tensile tests emphasized the excellent mechanical resilience of PGSA elastomers under periodic load (Figure 8b). The insertion of liquid metal conductive traces into PGSA provided an elastic electronic platform with ability to withstand up to 90% linear strain. Chen et al. reported 3D printed triboelectric nanogenerators (3DP‐TENGs) with 3D porous structures (Figure 8c).^[^ ^92^ ^]^ The PGS elastomer served as the elastic base material and one triboelectric material. MWCNTs were dispersed to form a conductive network and served as another triboelectric material. Micrometer‐sized salt particles were mixed with them to prepare composite “printing ink,” which played the role of thickener to ensure the rapid solidification. Subsequently, the salt particles played the role of enhancer to preserve the printed 3D structures in thermal‐cross‐linking process. After that, salt particles were easily removed by water to yield porous structure. The resultant 3DP‐TENG showed elastic modulus of 430 ± 60 kPa, which can be easily deformed by biomechanical motion. Neglectable hysteresis was observed in strain–stress curves according to cyclic compressive tests. 3DP‐TENG exhibited a maximum instantaneous power density of 1.11 W m^−3^ at an external load resistance of 50 MΩ. The stable output performance of 3DP‐TENG withstood more than 6000 cycles was also demonstrated (Figure 8d). After use, PGS elastomer could be completely degraded under enzyme catalysis. MWCNTs were also easily recycled and reused, and their electrical properties were well retained.

Besides the poor processability of PGS elastomers, the relatively low strength of PGS elastomers also severely hinders their large‐scale application. To improve the toughness and strength of PGS elastomers, several strategies have been developed, including increasing the cross‐linking densities, adjusting the molecular ratios of monomers, and incorporating nanofillers.^[^ ^93^, ^94^, ^95^, ^96^, ^97^, ^98^, ^99^, ^100^, ^101^, ^102^, ^103^ ^]^ Despite the considerable progress of enhanced strength, these strategies also compromised the soft nature and overall toughness, leading to an increase of modulus and decrease of maximum elongation. You et al. reported a new synthetic strategy of acid‐induced epoxide ring‐opening polymerization between diglycidyl sebacate and sebacic acid. Based on this strategy, a refined version of PGS, named poly(sebacoyl diglyceride) (PSeD), was prepared and demonstrated superior properties.^[^ ^104^, ^105^ ^]^ Cured PSeD elastomers showed a highly improved tensile strength (1.83 MPa) and maximum elongation at break (409%) compared with PGS (0.5 MPa, 267%). Subsequently, Chen et al. reported PSeD‐U (PSeD‐graft‐2‐ureido‐4[1H]‐pyrimidi‐none unit (UPy)) elastomers with physical and covalent hybrid cross‐linking structures by introducing the hydrogen bonding interactions into the covalently cross‐linked networks. The PSeD‐U possessed soft while strong mechanical properties and skin‐like nonlinear stress–strain behavior (Figure 8e).^[^ ^106^ ^]^ The different response of hydrogen bonds and covalent bonds to strain was considered as the key point. The hydrogen bonds having a low response to external stress under small strain and were in the state of “mechanical invisibility,” which limited the influence on the modulus of the elastomer. With the increase of strain, the response degree of hydrogen bond increased, which released hidden length to dissipate energy and enhance the strength and toughness of elastomers. As shown in Figure 8f, compared with PSeD elastomers, the strength and toughness of optimized PSeD‐U elastomers increased 3 and 11 times, respectively, while keeping low modulus (0.64 MPa). The elastomer exhibited skin‐like tear resistance (3670 ± 86.6 J m^−2^), almost four times higher than the widely used elastomer polydimethylsiloxane (PDMS) (960 J m^−2^), which was helpful to improve the stability of the corresponding flexible electronics. The superior biocompatibility and biodegradability of PSeD‐U elastomers were demonstrated both in vitro and in vivo. The capacitive pressure sensors based on the PSeD‐U dielectric materials were prepared with good pressure sensitivity and short response time, indicating the potential of PSeD‐U elastomers in wearable electronics (Figure 8g). The hybrid cross‐linking strategy has found a way out of incorporation of nanofillers and increasing the cross‐linking densities to improve the mechanical properties of biodegradable elastomers.

#### 3.1.3 Polyester Elastomers: POC

**Figure 9:**
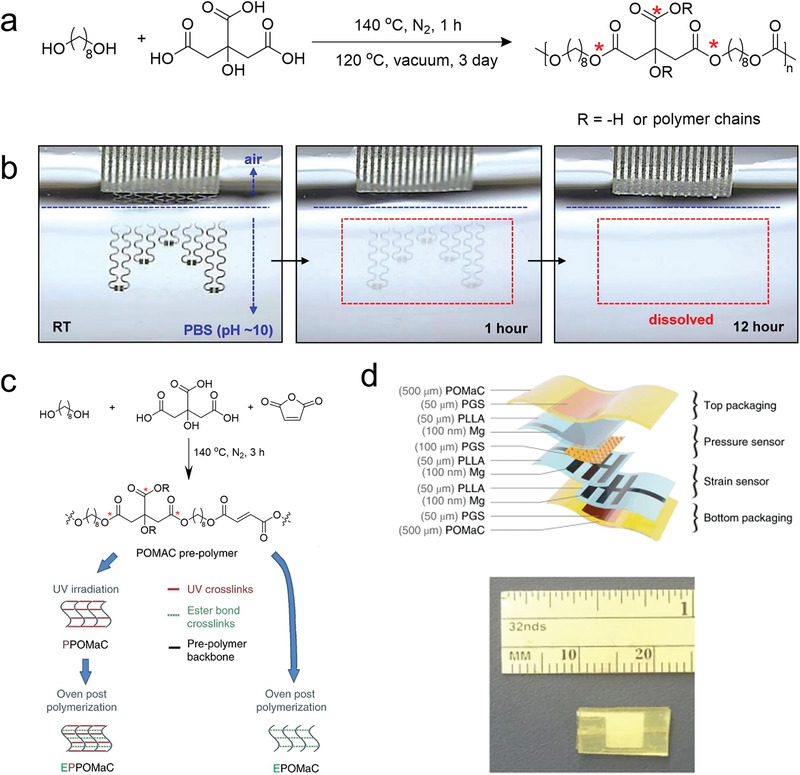
The POC elastomers used in electronics. a) Chemical structure of PGS, with emphasis on the susceptible sites (red stars) for biodegradability. b) Images at various stages of dissolution of a device during immersion in PBS. Reproduced with permission.^[^ ^109^ ^]^ Copyright 2015, American Chemical Society. c) Schematic diagram of chemical structures of POMAC with different synthesis path. d) Materials and overall assembly of the fully biodegradable strain and pressure sensor (top). Picture of the assembled sensor (bottom). Reproduced with permission.^[^ ^111^ ^]^ Copyright 2018, Springer Nature.

Another category of common biodegradable polyesters is diol/polybasic acid‐based elastomers, which have representative elastomers of POC.^[^ ^107^ ^]^ POC elastomers are first reported by Ameer's groups, which synthesized by directly thermally condensing 1,8‐octanediol with citric acid without the addition of any catalysts and cross‐linking reagents (**Figure** **9**a).^[^ ^108^ ^]^ Similar to PGS elastomers, the monomers of POC elastomers also have excellent biocompatibility and are safe for the body. The citric acid is a metabolic product of the Kreb cycle, while the 1,8‐octanediol is a water‐soluble and nontoxic aliphatic diol.^[^ ^68^ ^]^ Unlike the harsh cross‐linking condition of PGS elastomers, the POC prepolymers could form elastomers with mild conditions of even 37 °C under vacuum or ordinary pressure for times ranging from 1 day to weeks.^[^ ^108^ ^]^ POC elastomers exhibited desirable tissue‐like mechanical properties with Young's modulus ranging from 0.92 to 16.4 MPa, tensile strength of 2.6–6.1 MPa, and elongation at break of 117–265%, which are comparable with those of arteries and veins.^[^ ^107^ ^]^ The mechanical properties can be readily tuned by adjusting the molecular ratios of monomers and cross‐linking conditions. The increased cross‐linking temperature and time contributed to an increase of mechanical modulus and strength, but a decrease of elongation. The biodegradation of POC elastomers is also influenced by the cross‐linking densities, which higher degree of cross‐linking of networks leads to a lower biodegradation rate. Through the In vitro degradation experiments, the POC elastomers exhibited a complete degradation after 180 days when soaking in PBS at 37 °C. Owing to the enzymatic effects, the in vivo degradation of POC elastomers is usually faster than in vitro degradation.^[^ ^108^ ^]^

Rogers and co‐workers first introduced the POC elastomers into the fabrication of stretchable and transient devices as the substrate and encapsulant.^[^ ^109^ ^]^ The semiconductors (silicon nanoribbons), conductors (Mg), and dielectric material (silicon dioxide) were strain engineered on the surface of POC elastomers. With the serpentine interconnects ensuring the stretchability, the pH sensors and ion‐sensitive field effect transistor were prepared with functionality comparable to nonstretchable counterparts. The biodegradation of devices was investigated by immersing the devices in PBS (pH 10) at room temperature (Figure 9b). The Mg electrodes completely disappear after 12 h via reactive dissolution (i.e., hydrolysis), and other components (Si, SiO_2_, POC) dissolve slowly within several weeks. The photo‐crosslinked (poly(octamethylene maleate(anhydride) citrate)) (POMaC) was prepared to be used as encapsulation materials for in vivo sensors (Figure 9c,d).^[^ ^110^, ^111^ ^]^ The dually cross‐linked structures by both ultraviolet irradiation and polycondensation could enlarge the range of mechanical properties of POMaC elastomers with modulus between 0.03 and 1.54 MPa and maximum strains between 48% and 534%.

#### 3.1.4 Biodegradable Polyurethane Elastomers

**Figure 10:**
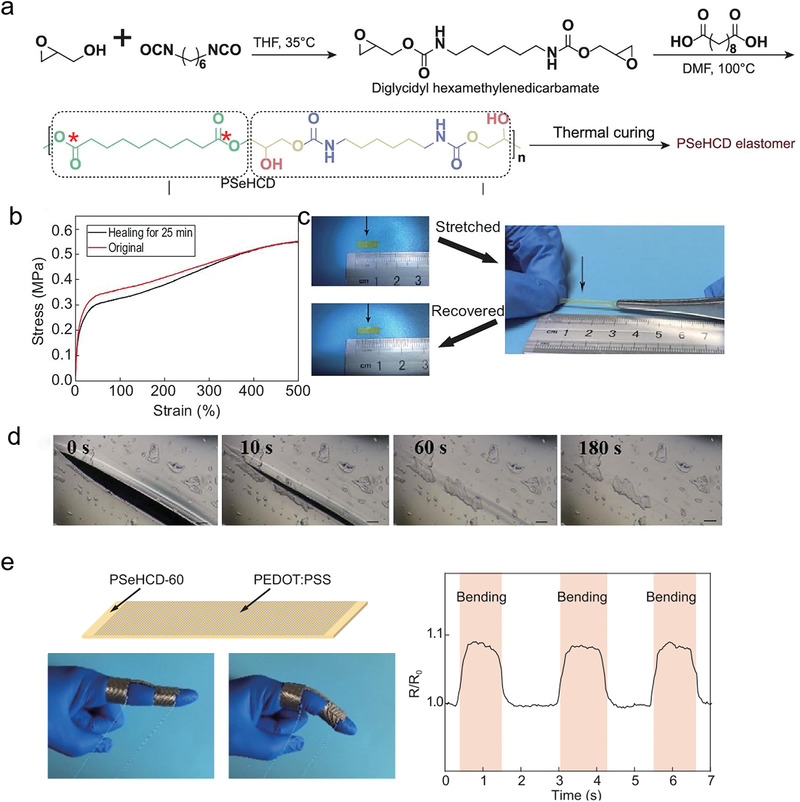
The biodegradable polyurethane elastomers. a) Schematic of the synthesis and structure of PSeHCD elastomers with both extensive evenly distributed H‐bonds physical cross‐linking and controlled partially chemical cross‐linking. The degradable sites were marked with red stars. b) Self‐healing and reprocessing of PSeHCD elastomers. Stress–strain curves of original and self‐healed PSeHCD‐60 strips. c) Optical images of the healed PSeHCD‐60 strip before and after stretching. d) Microscopic images of self‐healing procedure of a scratch on the surface of a PSeHCD‐60 film. e) The application of strain sensor based on PSeHCD‐60 and PEDOT:PSS. Reproduced with permission.^[^ ^116^ ^]^ Copyright 2021, Oxford University Press.

Recently, the newly developed elastomers with self‐healing properties have demonstrated their potential in the field of wearable electronics.^[^ ^112^, ^113^ ^]^ The electronic devices based on the self‐healing polymers could be robust and durable to withstand mechanical damage, which tremendously improves the stability of wearable electronic devices. For that purpose, the self‐healing biodegradable elastomers have been developed recently and attracted increasing attention. In the beginning, the self‐healing biodegradable elastomers were mainly based on the hydrogen bonds. Chen et al. first reported a new dynamic elastomeric network, where reversible hydrogen bonds instead of permanent covalent bonds were used to link the polymer chains together.^[^ ^114^ ^]^ UPy with self‐complementary quadruple hydrogen bonding was selected as the motifs to build dynamic polymer due to its suitability to build biomaterials. Because of the dynamic nature of hydrogen bonds, the resultant elastomers exhibited self‐healing properties under mild conditions and could be easily processed into various shapes using heat, pressure, and solvents. Almost at the same time, Guo and co‐workers also reported the PGS‐UPy based self‐healing elastomers.^[^ ^115^ ^]^ Owing to the strong hydrogen bonds of UPy motifs, these elastomers needed extra heat to self‐heal, which hinders their application in wearable electronics. The pioneering works still pave the way for the development of follow‐up related research. Very recently, inspired by the structures of peptidoglycan in bacterial cell walls, Zhang et al. reported designed a alternating polyester‐*co*‐polyurethane elastomer, poly (sebacoyl 1,6‐hexamethylenedicarbamate diglyceride) (PSeHCD) elastomer, with chemically and physically hybrid cross‐linking (**Figure** **10**a).^[^ ^116^ ^]^ The alternating ester groups enabled steady biodegradation performance, while the alternating urethane groups introduced extensive and evenly distributed hydrogen bonds, leading to an autonomous self‐healing performance at room temperature without extra stimulation. As shown in Figure 10b,c, when the PSeHCD‐60 strip was cut into two pieces and slightly rejoined at room temperature for 25 min, the self‐healed strip showed almost similar tensile behaviors to the original and comparable toughness (4.05 MJ m^−3^) at over 90% of the original (4.42 MJ m^−3^). In the surface scratch self‐healing experiment, a scratch of 100 µm could completely be healed within 3 min at 15 °C (Figure 9d). Strain sensors prepared based on the PSeHCD and PEDOT:PSS exhibited higher strain sensitivity and could effectively monitor the bending motion of fingers (Figure 10e). The strain sensor that was completely cut off and healed for 2 min at room temperature, showed a resistance‐based dynamic response similar to that of the original intact sensor in the cyclic tensile test. More biodegradable elastomers based on physical cross‐links have been reported and would greatly promote the development of the field of self‐healing biodegradable elastomers.^[^ ^117^ ^]^ Besides, a thermoset polyurethane elastomer based on Cu(II)–dimethylglyoxime–urethane complex with both high efficiency in self‐healing and excellent mechanical properties at ambient conditions was reported.^[^ ^118^ ^]^ The copper ions not only coordinated with oxime groups to improve the strength and toughness but also facilitated the dynamic exchange of oxime–urethane bonds. Liquid metals were used to composite with elastomers to construct conductors. The severed composite conductor was efficiently healed within 9 min under ambient conditions, which could light the light‐emitting diode (LED) on even while being stretched to 250%. More importantly, the biodegradability of this elastomer was demonstrated by in vivo tests, which originated from the dynamic nature of oxime–urethane groups.^[^ ^119^ ^]^ This novel type of biodegradable group would offer more choices for the construction of new electronic elastomers with high performance and promote the development of the field of biodegradable elastic electronics.

#### 3.1.5 Dynamically Covalent Cross‐Linked Elastomers

**Figure 11:**
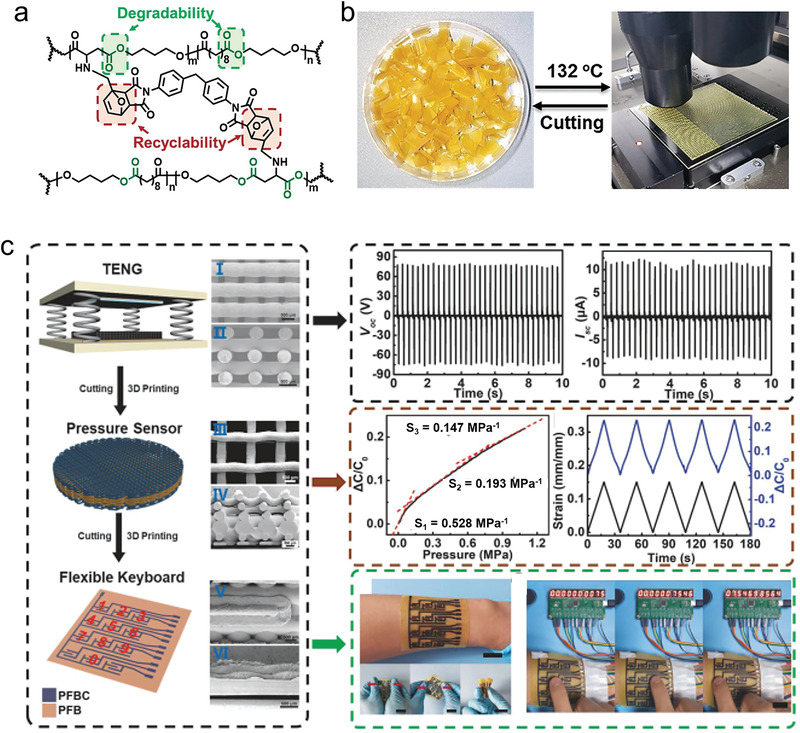
The dynamically covalent cross‐linked elastomers. a) Molecular structure of the PFB with dynamic covalent bond by DA reaction. The degradable sites were marked with green blanks. b) The 3D printing of the PFB elastomer. c) The recycle of electronics based on PFB elastomers. From top to bottom: TENG, pressure sensor, and flexible keyboard (right) and the corresponding electrical performance (left). Reproduced with permission.^[^ ^120^ ^]^ Copyright 2020, Wiley‐VCH.

Except for the noncovalent interactions, the dynamic covalent bonds were also introduced into the construction of biodegradable elastomers to obtain the self‐healing and recycling properties. Guo et al. developed a new electronic dynamically covalent cross‐linked elastomer, named PBSF‐FA‐BMI (PFB) based on the Diels–Alder reaction (**Figure** **11**a).^[^ ^120^ ^]^ Based on the reasonable molecular structure design, the PFB elastomers showed a favorable combination of elasticity, biodegradability, processability, and recyclability. The PBSF‐FA‐2BMI (furan/maleimide = 3/2) showed a maximum elongation of 350%, modulus of 1.87 ± 0.08 MPa, and tensile strength of 1.9 ± 0.28 MPa, as well as the stability to heat, chemicals, and humidity. The durability of intermediates of the DA reaction to the moisture and oxygen makes the 3D printing of PFB elastomers available (Figure 11b). The elastomers exhibited clearly rheological change from thermoset to thermoplasticity during the heating process. The conductive fillers including CNTs, Ag nanoflakes, and carbon black were used to fabricate elastic conductive composites (PFBC) for electronics. The mechanical and conductivity of PFBC composites showed good retention during the recycling. Owing to the photothermal effect of nanofillers, the PFBC composites could completely self‐heal after irradiating for 1 min using near‐infrared light. As presented in Figure 11c, different types of electronic devices, including TENG, pressure sensor, and flexible keyboard were fabricated with stable electrical performance, demonstrating their recyclability. Besides, 3DP‐flexible keyboard degraded ≈15% in 1 month by soaking in lipase enzyme solutions, demonstrating the biodegradability of PFB‐based electronic devices.

### 3.2 Biodegradable Elastomer‐Based Conductors and Semiconductors

Organic conducting and semiconducting elastomers would be ideal candidates for the next‐generation fully organic elastic electronics.^[^ ^23^ ^]^ The conjugated polymers, such as polypyrrole, polyaniline, and PEDOT are not only widely used as electronic circuits to connect various components, but also collect electrical signals in vivo, such as neurons and cardiac tissues.^[^ ^121^ ^]^ Conjugated polymers can be either semiconducting or conducting, depending on their Fermi level, which has gained a prominent position in the fabrication of flexible and conformal electronic devices due to their stable electronic conductivity and high mechanical flexibility. Considering the non‐biodegradable and rigid nature of conjugated polymers, it remains challenging to endow the biodegradability and elasticity to conductors and semiconductors.^[^ ^122^ ^]^ Previous works have demonstrated that short‐range intermolecular aggregation was sufficient for efficient long‐range charge transport and provided high mobility and flexible properties could be realized simultaneously in macroscopically disordered conjugated polymers with interconnected aggregates.^[^ ^123^ ^]^ Based on this mechanism, strategies for the fabrication of biodegradable conductors and semiconductors were developed and remained in two categories: co‐polymerizing or compositing with biodegradable components.^[^ ^26^ ^]^

**Figure 12:**
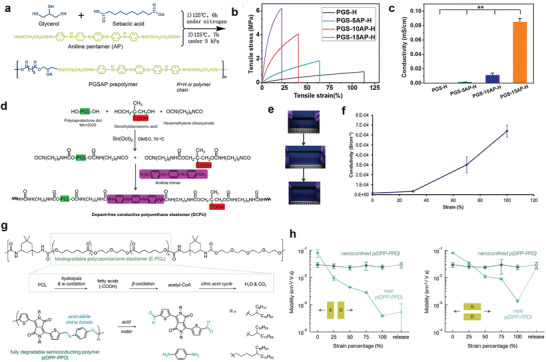
The biodegradable elastic conductors and semiconductors. a) Schematic synthesis of PGSAP prepolymer and cross‐linking with HDI to obtain PGSAP‐H polymers. b) Stress–strain behavior of PGSAP‐H polymer films. c) The conductivity of PGSAP‐H polyurethane films. Reproduced with permission.^[^ ^62^ ^]^ Copyright 2016, Elsevier. d) Synthetic scheme of the DCPU. e) Biodegradable DCPU film and its high elasticity presented by stretching and recoiling. f) Dependence of electrical conductivity of DCPU‐0.3/1 on applied strains. Reproduced with permission.^[^ ^124^ ^]^ Copyright 2016, Springer Nature. g) Chemical structure of the biodegradable elastomer (E‐PCL) based on polycaprolactone and the known degradation pathway of PCL (upper). Chemical structure of the fully degradable semiconducting polymer, p(DPP–PPD), and the monomeric byproducts after initial cleavage (bottom). h) The change in saturation mobility of neat and nanoconfined p(DPP–PPD) during stretching to 100% strain with both parallel (left) and perpendicular (right) to the charge transport direction. Reproduced with permission.^[^ ^61^ ^]^ Copyright 2019, American Chemical Society.

Ma and co‐workers reported conductive biodegradable flexible polyurethane through the polycondensation of PGS and aniline pentamer (**Figure** **12**a).^[^ ^62^ ^]^ As shown in Figure 12b, the flexible segments of PGS provided degradability and elasticity to the copolymers with strength ranging from 1.7 to 5.3 MPa and strain range from 17% to 55%. The conductivity prepared polyurethanes doped with camphorsulfonic acid were in the range from 1.4 × 10^−6^ to 8.5 × 10^−5^ S cm^−1^ (Figure 12c). The elastic nature involved with conductivity effectively enhanced Schwann cells’ myelin gene expressions and corresponding neurotrophin secretion. Figure 12d presented a dopant‐free conductive polyurethane elastomer (DCPU) by chemically linking biodegradable segments of PCL, conductive segments (aniline trimer), and dopant molecules (dimethylolpropionic acid (DMPA)).^[^ ^124^ ^]^ DCPU showed decent elasticity, demonstrating by instant recovery (>99%) after three cycles of stretching at 10% strain (Figure 12e). The dry DCPU possessed conductivities ranging from 10^−8^ to 10^−5^ S cm^−1^. The conductivities of DCPU displayed a unique strain‐induced increase phenomenon (43‐fold increase when stretched to 100%), which was primarily due to the oriented polymer chains (Figure 12f). DCPU showed hydrolysis and enzymatic degradation behaviors. The degradation rate was influenced by the DMPA concentration, which could increase the penetration ability of water to the DCPU.

For the degradable semiconductors, Bao's group reported a breakthrough work using hydrolyzable and reversible imine bonds as conjugated linkages between diketopyrrolopyrroles (DPP) and p‐phenylenediamine (PPD) under catalysis with p‐toluenesulfonic acid to develop fully disintegrable and biocompatible semiconducting polymers.^[^ ^125^ ^]^ Based on this research, Tran et al. composited the degradable semiconductors with urethane‐based elastomers featuring biodegradable E‐PCL to construct the stretchable and fully degradable semiconductors (Figure 12g).^[^ ^61^ ^]^ At 70 wt% of E‐PCL, the composites maintained a reasonable combination of mobility (≈0.05 cm^2^ V^−1^ s^−1^) and minimized the conjugated polymers content. The composites showed no change in mobility (≈0.03 cm^2^ V^−1^ s^−1^) at strains up to 100% and subsequent release, without thin‐film crack formation (Figure 12h). The resultant semiconductive elastomers could degrade in the acidic aqueous solutions (containing 1 m trifluoroacetic acid). Although the overall properties of the conductive and semiconductive elastomers have the room to improve for practical applications, these works pave a new perspective to construct full biodegradable electrical active elastomers. On the basis of these pioneering works, it can be predicted that the future advances of biodegradable functional elastomers would powerfully push the development of wearable and implantable electronic devices.

### 3.3 Biodegradable Gels

Gels are kind of materials with 3D polymeric network structures, which could swell in solvent and can keep a large volume of solvent without dissolving. The interactions among the polymer chains are vital for the formation of gels, including physical entanglement of polymer chains, hydrogen bonding, *π*–*π* stacking, electrostatic interactions, and covalent cross‐linking.^[^ ^126^ ^]^ The aggregate state of gels is neither completely solid nor completely liquid, but an intermediate state of matter between them. The solid behavior of gels is to maintain their shape and volume under certain conditions, while the behavior of liquids is that solvent can diffuse or permeate from gels. Thus, the gels could retain the chemical and physical properties of liquid with maintaining the dimensional stability of a solid, which makes the gels work as intrinsic ionic conductors available. Depending on the types of solvents, the gels can be divided into hydrogels (water), organogels (organic solvents), organohydrogels (binary solvent of organic solvent and water), and ionic gels (ionic liquid). Among these, hydrogels are most widely used due to their excellent biocompatibility and facile preparation.^[^ ^127^, ^128^, ^129^, ^130^ ^]^ As an important class of soft matter, the hydrogels behave as a solid and are mechanically viscoelastic in nature. Similar to elastomers, hydrogels also possess superior mechanical elasticity, which can be stretched several times their initial length and recover completely. The Young's modulus of most hydrogels is also close to those of soft tissues, ranging from 1 to 100 kPa.^[^ ^64^ ^]^ The elasticity of hydrogels is essentially entropy elasticity, resulting from the conformational change of polymer chains. Compared with electronic conductors, the deformation of hydrogels exhibits less effect on their ionic conductivity. Besides, most of the hydrogels are transparent to all colors of visible light, which is another important characteristic compared with electronic conductors. The polymer networks in the hydrogels does not scatter light, which enable the hydrogels retain optical properties of transparency as the thickness increases, similar to water.^[^ ^64^ ^]^ The comprehensive properties of hydrogels make them ideal materials for elastic electronics applications, such as wearable electronics and implantable human–machine interfaces.

Among the reported hydrogels, natural hydrogels are recognized as the biodegradable and biocompatible candidate for elastic electronics.^[^ ^54^, ^131^, ^132^ ^]^ There are two kinds of widely used natural hydrogels: polysaccharides (e.g., cellulose, chitosan, alginate) and protein‐based polymers (e.g., silk, gelatin). Considering this review article focuses on the elastic hydrogels for electronic application, the chemical structure, properties, and corresponding applications in elastic electronics of alginate and gelatin‐based hydrogels and their corresponding organohydrogels would be discussed in detail and the obtained results are summarized in **Table** **2**. Details about the applications of the natural polymers of cellulose, chitosan, and silk for stretchable conductors and electronics have been highlighted in other recent reviews.^[^ ^133^, ^134^, ^135^ ^]^

**Table 2:** Summary of recent advances in the development of the alginate‐ and gelatin‐based hydrogels for electronic applications

Materials	Potential component in devices	Remarkable results
Alginate/PAAm	Substrates and conductors	Constructing double networks leads to ultrahigh stretchability and fracture toughness.^[^ ^139^ ^]^
Ecoflex‐alginate/PAAm	Substrates	The hybrid of elastomers and hydrogels enables the interfacial compatibility with conductive inks, while skin‐like modulus and large stretchability.^[^ ^140^ ^]^
Sodium alginate nanofibrillar/PAAm	Substrates and conductors	The assembled sodium alginate nanofibrillar and cross‐linked PAAm form dermis‐mimicking structures, leading to excellent mechanical properties and ionic conductivity.^[^ ^150^ ^]^
Organohydrogel fibers	Conductors	Wet‐spinning strategy enables continuous fabrication of conductive organohydrogel fibers with excellent antifreezing (<−80 °C), stability (>5 months), transparency, and stretchability.^[^ ^155^ ^]^
Gelatin‐based biogels	Substrates and conductors	Resilient yet sustainable single platform with self‐adhesion and self‐healing properties for robots and electronics.^[^ ^162^ ^]^
Gelatin–ammonium sulfate hydrogels	Substrate and conductors	Significant improvement of strength and stretchability of gelatin hydrogels based on the Hofmeister effect.^[^ ^173^ ^]^
Gelatin organohydrogel	Substrates and conductors	The interactions between gelatin and sodium citrate as well as solvent of glycerine/water endow the organohydrogel with high strength, ionic conductivity and environment tolerance.^[^ ^176^ ^]^
GelMA	Substrates and conductors	The introduction of methacryloyl endows the gelatin with photo‐crosslinking properties.^[^ ^177^ ^]^

#### 3.3.1 Alginate‐Based Gels

Alginate is a kind of polysaccharide polymer derived from brown seaweeds, which is known for its appealing unique properties, including superior biocompatibility, biodegradability, processability, as well as their mass production with relatively low cost.^[^ ^136^, ^137^ ^]^ Alginate‐based materials have been widely used both in the field of industries and scientific research, such as the food industry, pharmaceutical industry, cosmetic industry, tissue engineering, and drug delivery. Alginate is a polyanionic linear block copolymer containing blocks of (1‐4)‐linked *β*‐d‐mannuronic acid (M) and *α*‐l‐guluronic acid (G).^[^ ^138^ ^]^ The G‐block in alginate tends to coordinate with the multivalent cations (e.g., calcium, magnesium) to form the physical cross‐linking, by which the alginate solutions (e.g., sodium alginate solution) could easily form the hydrogels under ambient conditions.^[^ ^139^ ^]^ The physical and chemical properties of alginate hydrogels are vastly dependent on the ratios of these two uronic acids and the corresponding sequential arrangements.^[^ ^138^ ^]^ The higher G‐block concentration increases the cross‐linking density, resulting in rigid mechanical properties, whereas the higher M‐block content makes the hydrogels to be soft. A large amount of hydroxyl and carboxyl groups in the alginate molecular chain give alginate an attractive ability of chemical modification, which is intriguing to endow the desired properties to alginate hydrogels.^[^ ^54^ ^]^ Various processing strategies have been developed for the alginate hydrogels based on the rapid and facile cation‐induced gelation. For the electronic applications, the alginate hydrogels are widely used as substrates and conductors due to their water solubility, easy processing, commercial availability, and bio‐friendly properties.^[^ ^54^ ^]^

**Figure 13:**
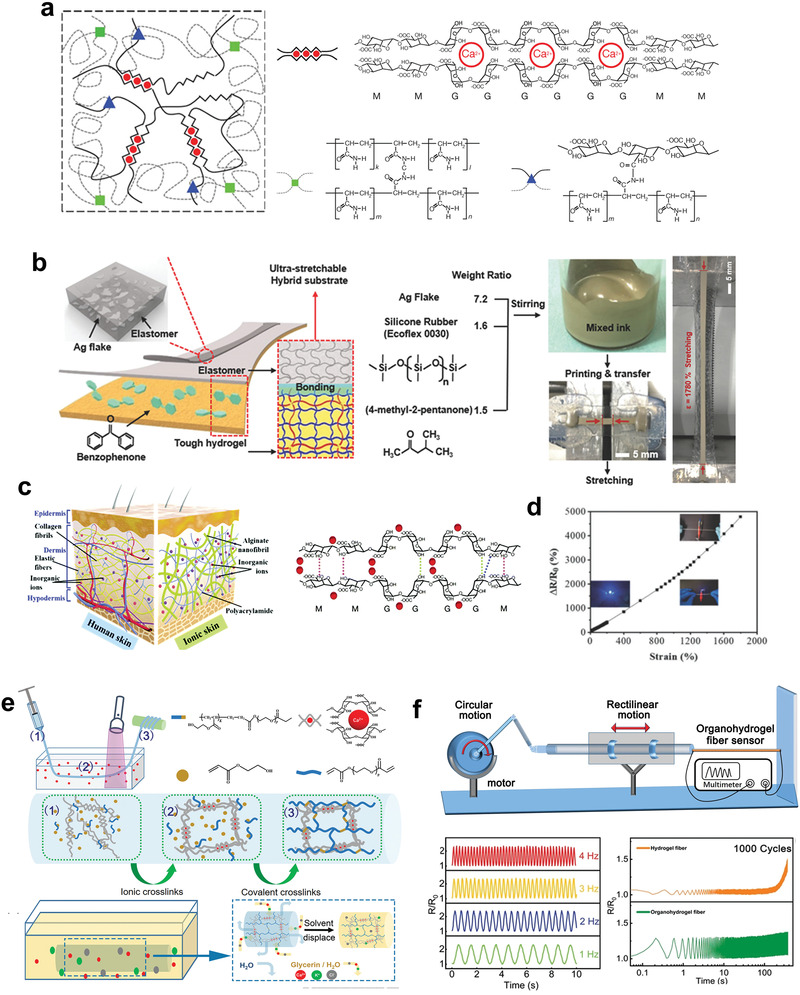
The alginate‐based gels. a) Schematic diagram of the alginate–polyacrylamide hybrid gel with double networks. Reproduced with permission.^[^ ^139^ ^]^ Copyright 2012, Springer Nature. b) The fabrication and photographs of the stretchable conductor containing the hybrid substrate and conductive ink layer. Reproduced with permission.^[^ ^140^ ^]^ Copyright 2018, Wiley‐VCH. c) Design and synthesis of high‐performance sodium alginate (SA)/NaCl/PAAm ionic double network hydrogels. d) Relative resistance changes of the sensors on applied tension. The inset pictures are the luminance variations of LEDs with the increase of tensile and compression loading. Reproduced with permission.^[^ ^150^ ^]^ Copyright 2019, Royal Society of Chemistry. e) Design and fabrication of organohydrogel fibers. f) The application of organohydrogel fiber as a sensor for high‐frequency and high‐speed motion. Reproduced with permission.^[^ ^155^ ^]^ Copyright 2020, Wiley‐VCH.

For the practical applications, the alginate hydrogels have desired skin‐like modulus but poor stretchability and tear resistance. To improve the mechanical performance of alginate hydrogels, strategies of interpenetrating networks of ionically cross‐linked alginate networks and long‐chain covalently cross‐linked polymer networks have been developed. The ionically cross‐linked networks act as the sacrificial networks to dissipate significant applied energy, while the covalently cross‐linked networks enable the integrity under large deformations. Sun et al. introduced the polyacrylamide (PAAm) as the second network into the alginate networks cross‐linked by Ca^2+^, leading to ultrahigh stretchability(≈2100% strain) and fracture toughness (≈9000 J m^−2^), demonstrating the effectiveness of this strategy for the improvement of stretchability, toughness, and robustness (**Figure** **13**a).^[^ ^139^ ^]^ Therefore, various tough alginate‐based hydrogels have been vastly prepared and studied as stretchable substrates of electronics for wearable applications.^[^ ^54^ ^]^ Kim et al. reported a strategy for fabricating printable and highly stretchable conductors based on tough alginate‐based hydrogels (Figure 13b).^[^ ^140^ ^]^ The hybrid substrates were composed of two layers of thin‐film Ecoflex and tough hydrogels. Benzophenone was used as bonding agent to ensure the strong adhesion between hydrogels and Ecoflex by forming covalent bonds. The Ecoflex film improved the interfacial compatibility of hydrogel with conductive inks, leading to elongation at break of 1780% with decent conductivity. The patterned conductors as the wiring line for the LED even worked under a tensile strain of 200%. The skin‐like modulus and large stretchability of alginate hydrogels enabled the hybrid substrates as proper materials to easily and comfortably attach to human skin, indicating the potential for the wearable electronics. Furthermore, electronically conductive hydrogels have been fabricated by a one‐step deposition process by directly transferring the single‐walled carbon nanotube films onto the hydrogel without the need for a sacrificial layer or any other intermediate steps.^[^ ^141^ ^]^ The resultant conductors could withstand intrinsic stretching up to 100% strain. The reported mechanically interlocked hydrogel—elastomer hybrids based on tough alginate hydrogels also demonstrated their potential for skin‐like electronics.^[^ ^142^, ^143^ ^]^

Except used as substrates, the alginate hydrogels can also be utilized as biocompatible and biodegradable conductors.^[^ ^54^ ^]^ Alginate hydrogels have decent intrinsic ionic conductivity or can be used as matrix compositing with conductive fillers to obtain superior electronic conductivity. The soft, tough, and robust nature of alginate‐based hydrogels enables the large range of applications, which is vastly important for the application of elastic biocompatible electronics. Like elastomers, homogenous dispersing the conductive fillers in the alginate solutions, to form the percolation conductive networks, is the most representative method to fabricate the biocompatible, biodegradable, and elastic conductors. The abundant polar groups on the alginate chains can form interactions with charged groups of conductive fillers, which effectively improves the stability of the composite systems. Typical conductive hydrogel composites were prepared by mixing AgNWs with sodium alginate aqueous solution and then cross‐linked with Ca^2+^.^[^ ^144^ ^]^ AgNWs showed good metallic conducting filler materials due to their excellent electrical conductivity, ultralong 1D structure, and scale synthesis. The resultant conductive composites exhibited high conductivity and elasticity, which also can be processed into various shapes by laser cutting. Except for 1D nanomaterials, 2D conductive nanomaterials have also been incorporated as fillers to prepare the conductive composites. Wu et al. reported a conductive, self‐healing, adhesive, and long‐lastingly moist Mxene nanocomposite hydrogel by coating of the Mxene nanosheet network using the polymer networks of dopamine grafted sodium alginate (Alg‐DA), phenylboronic acid grafted sodium alginate (Alg‐PBA), and PAAm with a glycerol/water binary solvent as the dispersion medium.^[^ ^145^ ^]^ Mxene was used as filler due to its high specific surface area, excellent hydrophilicity, and good conductivity. The formation of dynamic covalent ester bonds between the PBA group of Alg‐PBA and the DA group of Alg‐DA enable the cross‐linking structure of the hydrogel. The resultant hydrogel showed long‐term stability (10 days), which was attributed to the strong hydrogen bonds between water and glycerol hindering the water evaporation. Besides, the hydrogel exhibited excellent self‐healing properties due to the dynamic PBA—catechol bonds. The existence of the catechol groups of PDA and the hydroxyl group of glycerol, the hydrogels exhibited excellent self‐adhesive capability to different surfaces, which can be comfortably integrated with human skins for wearable epidermal sensors. Beyond the mentioned conductive nanomaterials, other homogenous dispersed conductive nanomaterials or conjugated polymers including graphene oxide (GO), MWCNTs, PEDOT:PSS, and liquid metals were also used as fillers to composite with alginate hydrogels to develop the biodegradable, biocompatible, and elastic conductive composites for electronics.^[^ ^146^, ^147^, ^148^, ^149^ ^]^

The water‐soluble properties and ion‐induced fast gelation of alginate make the excellent ionic conductivity of hydrogels available. Various formats of alginate hydrogels conductors have been developed based on the ionic conductivity. Zhang et al. reported a new class of bio‐inspired ionic conductors based on supramolecular sodium alginate nanofibrillar and chemically cross‐linked PAAm hydrogels (Figure 13c).^[^ ^150^ ^]^ Particularly, the salts (e.g., NaCl) were used as triggers for supramolecular assembly of sodium alginate to form the nanofibrils structures, which simulated the collagen fibers in the dermis. Meanwhile, the PAAm acted as the 3D cross‐linked elastic elastin to provide superior softness and elasticity. Owing to the dermis‐mimicking structures, these hydrogels exhibited excellent mechanical properties with a tension strength of 4 MPa, stretchability of 3120%, the toughness of 4.77 MJ m^−3^. Moreover, the resultant hydrogels also showed strain‐dependent conductivity, which increased nearly linearly with the increase of strain (Figure 13d). The potential application of hydrogels was demonstrated as strain sensors with high sensitivity to an extremely broad strain window (0.3 — 1800%) and a low applied voltage (down to 0.04 V), as well as high‐pressure sensitivity (1.45 kPa^−1^). In addition, bioinspired mineral alginate‐based hydrogels were prepared and exhibited broad prospects in the field of biomedical electronics.^[^ ^151^ ^]^

For the practical application, the freezing and evaporation of water in the hydrogels leads to dysfunction, and thus seriously hampers their use in subzero or dry environments. To broaden the range of working environments of hydrogel‐based devices, organic liquids (e.g., glycerol and ethylene glycol) and antifreezing agents (e.g., zwitterionic) have been introduced into conductive hydrogels to possess the improved antifreezing capability and long‐term stability.^[^ ^152^, ^153^, ^154^ ^]^ Alginate/PAAm tough hydrogels with decent antifreezing properties were easily prepared by adding a suitable amount of an ionic compound.^[^ ^153^ ^]^ After soaking hydrogels into CaCl_2_ aqueous solutions, the resulting hydrogels cooled to temperatures as low as −57 °C without freezing and while it kept excellent elasticity with a fracture toughness of 5000 J m^−2^. Considering the development trend of lightweight and breathability for wearable electronics, hydrogel fibers with excellent ionic conductivity and elasticity would be an important platform for the next‐generation electronics. Developing continuous and scale production of hydrogel fibers still remains challenging due to the poor spinnability of hydrogels and their precursor solutions. Song et al. reported an efficient strategy based on wet spinning to fabricate a new type of conductive fibers with highly stretchability, transparency, antifreezing, durable ionic conductivity (Figure 13e).^[^ ^155^ ^]^ The precursor solution containing sodium alginate, polyethylene glycol diacrylate (PEGDA), and 2‐hydroxyethyl acrylate (HEA) was spun into a Ca^2+^ aqueous solution under ultraviolet light. In the coagulation bath, alginate and Ca^2+^ rapidly form an ionic‐crosslinked nascent fiber, which tolerated the drafting force before the formation of a covalently cross‐linked network. The PEGDA was used to improve the extrudability of precursor solutions, while the HEA worked as a stabilizer to improve the interaction between polymers and solvents. Afterward, the corresponding organohydrogel fibers were prepared by simple solvent replacement of as‐prepared hydrogel fibers. The organohydrogel fibers showed excellent antifreezing (<−80 °C), stability (>5 months), transparency, and stretchability. The predominantly covalent cross‐linked network endowed the fiber's high dynamic mechanical stability with negligible hysteresis and creep, from which previous conductive fibers usually suffer. Accordingly, strain sensors made from organohydrogel fibers accurately captured high frequency (4 Hz) and high‐speed (24 cm s^−1^) motion and exhibited little drift for 1000 stretching—releasing cycles, which were powerful to detect rapid cyclic motions such as the valve of the engine and difficult to reach by previously reported conductive fibers (Figure 13f). The organohydrogel fibers also demonstrated potentials as the wearable anisotropic sensor, data gloves, soft electrodes, and optical fibers.

#### 3.3.2 Gelatin‐Based Gels

Gelatin, as an edible, degradable, and bio‐based polypeptide has been widely used in food production, cosmetics, and medications due to its excellent biodegradability, biocompatibility, water‐solubility, easy processability, naturally abundant, and low cost.^[^ ^156^ ^]^ Gelatin is prepared by the hydrolysis of natural animal collagen, which is the most abundant protein in animals, featuring three long helix‐shaped chains of amino acids.^[^ ^157^ ^]^ Depending on the different pretreatment processes, the gelatins are divided into two main types: type A gelatin (acid hydrolysis) and type B gelatin (alkaline hydrolysis). The physical and chemical properties of gelatin are vastly affected by the pretreatment process (isoelectric point 8–9 for type A, isoelectric point 4–5 for type B) and amino acid composition (depending on collagen source).^[^ ^158^ ^]^ Gelatin hydrogels could be easily prepared by dissolving in hot water and cooling to room temperature. The gelatin‐based hydrogels exhibit excellent biocompatibility and biodegradability, which are used in vivo with diverse fields.^[^ ^159^, ^160^, ^161^ ^]^ The gelation process is related to the transition of gelatin polymer chains between random coil structure and partially restored triple helix structure, which is a special thermos‐reversible process. The triple‐helix structures of gelatin result from the interchain hydrogen bonds between carbonyl and amines groups on polypeptide chains. At high temperatures, the triple‐helix structures are broken and gelatin polypeptide chains show a random coil configuration, leading to good solubility in aqueous solutions. With temperature decrease, the polypeptide chains interact to form a partial collagen‐like triple helix, resulting in gelation at room temperature. Depending on the concentration and type of gelatin, the gelation temperature floats up and down around room temperature. Different from the poor stretchability and strength of pure alginate hydrogels, the gelatin hydrogels exhibited a combination of superior mechanical properties and biodegradability as well as biocompatibility, making them excellent potential candidates as substrates or conductors for elastic bio‐friendly electronics.

**Figure 14:**
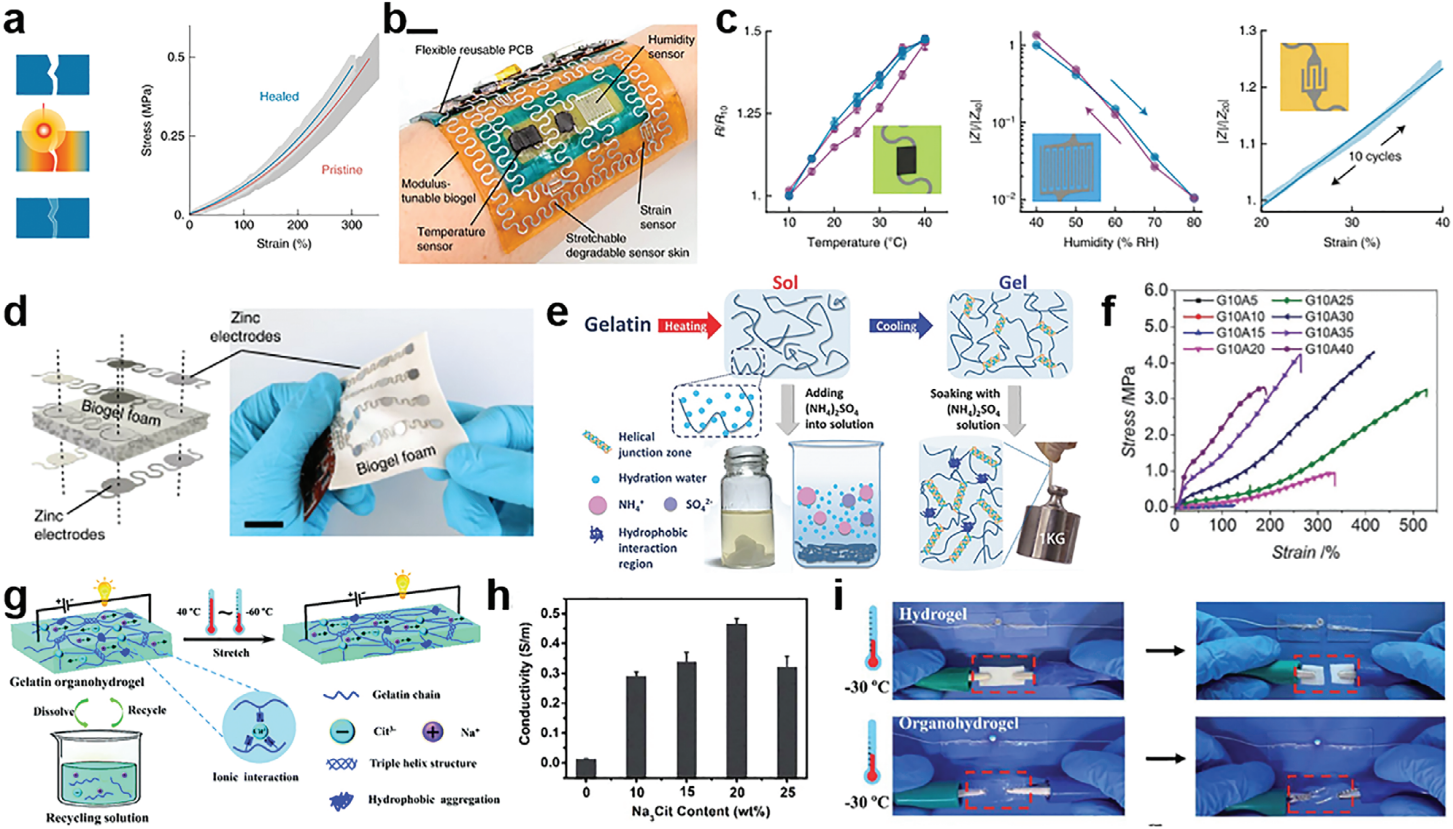
The gelatin‐based gels. a) Schematic of the laser healing process (left) and the corresponding stress–strain curves of pristine and cut‐and‐healed gel samples (right). b) Soft e‐skin on a human arm. Scale bar = 3 cm. c) Characterization of the temperature sensors (left), humidity (middle), and strain sensors (right) of the soft e‐skins. d) Schematic illustration of orientation and design of zinc electrodes and the degradable stretchable pressure sensor array based on biogel foam. Scale bar = 2 cm. Reproduced with permission.^[^ ^162^ ^]^ Copyright 2020, Springer Nature. e) Strengthening mechanisms of gelatin–ammonium sulfate hydrogels. f) Tensile stress–strain curves of the gelatin hydrogels with different (NH_4_)_2_SO_4_ concentrations. Reproduced with permission.^[^ ^173^ ^]^ Copyright 2017, Wiley‐VCH. g) Schematic illustration of the structure and versatility of the fully recyclable gelatin organohydrogel. h) The influence of Na3Cit amounts to the conductivity of the organohydrogels. i) The conductivity and mechanical robustness of the hydrogel and organohydrogel after freezing at 30 °C for 24 h. Reproduced with permission.^[^ ^176^ ^]^ Copyright 2020, Royal Society of Chemistry.

A versatile gelatin‐based biogel that unites the challenging needs of resilient yet sustainable robots and electronics in a single platform was developed.^[^ ^162^ ^]^ The biogels derived entirely from natural and food‐safe constituents, possessing highly resilient with outstanding elastic characteristics, and could be fully degraded in several days after use. The additional bio‐derived components of citric acid and glycerin endowed the antibacterial properties and long‐term stability of biogels. By adjusting the interaction between food‐safe additives (sugar and glycerin) and the gelatin network, outstanding mechanical properties were obtained through systematic optimization. The resultant biogel showed a small hysteresis on repeated cyclic. Limited fatigue was found after thousands of cyclic tensile tests, yet no failure occurred after more than 100 000 cycles. The biogels showed self‐adhesion and self‐healing properties, which broaden their applications (**Figure** **14**a). Soft and degradable electronic sensor patches were also developed combined with biodegradable zinc metals as electrodes. Based on the laser‐assisted rapid self‐healing properties, the sensor patches with different functional modules easily integrated together without influence on the sensing capability (Figure 14b,c). The self‐adhesion nature of gelatin biogels provided good and comfortable wearability, confirming the potential in the skin‐like wearable electronics. Besides, the pressure‐sensitive e‐skin array from a 1‐mm‐thick biogel foam together with a 4 × 4 matrix of zinc‐foil electrodes with stretchable interconnects were prepared, demonstrating the expansibility of biogel (Figure 14d). This breakthrough work profoundly revealed the potential application of gelatin hydrogels in future biodegradable electronic devices that can avoid microplastics and are inherently safe when interacting with other lifeforms.

Except used as substrates, the gelatin hydrogels also show great advantages to be used as conductors for biodegradable elastic electronics. Like alginate hydrogels, the excellent water solubility of gelatin enables the availability for compositing with conductive nanomaterials, such as carbon‐based, metal‐based nanomaterials and conducting polymers to fabricate conductive composites based on gelatin matrix.^[^ ^163^, ^164^, ^165^, ^166^, ^167^, ^168^, ^169^, ^170^ ^]^ Abundant functional groups on the polypeptide chains of gelation provide diverse chemical modification sites, by which the physical and chemical properties can be easily tuned by different chemical compounds or cross‐linking agents. Besides, the dependable electrostatic charged nature and aggregation structures of polypeptides also enable different strategies of physical modifications, leading to gelatin‐based hydrogels with improved properties. A biocompatible and electrically conductive elastic hydrogels (GE‐SH‐AgNW) were prepared using gelatin as polymer matrix to composite1D Ag NWs.^[^ ^167^ ^]^ In order to improve the stability of conductive composites, the thiol groups were chemically introduced on the gelatin molecular chains for better interactions between the AgNWs and the gelatin matrix. Subsequently, the hydrogels were immersed in a 20 wt% Na_2_SO_4_ solution to introduce the additional physical crosslinks into composites by the “salting out” effect to improve the mechanical properties. The elongation of GE‐SH‐AgNW composite hydrogel reached 350%, and the tensile strength was 50% higher than that of the pure GE hydrogel. Moreover, its conductivity reached as high as 0.1 S cm^−1^, exhibiting great potential for the biodegradable elastic electronics. Using ions to improve the mechanical properties of gelatin hydrogel was demonstrated an effective strategy, which is mainly based on the Hofmeister effect. Hofmeister effect was first found by Hofmeister in 1888, which is an order of ions that have the ability to alter the solubility of proteins.^[^ ^171^ ^]^ The anions depending on their ability have been found in an order: CO_3_^2−^ > SO_4_^2−^ > H_2_PO^4−^ > F^−^ > CH_3_COO^−^ > Cl^−^ > Br^−^ > NO^3−^ > I^−^ > SCN^−^.^[^ ^172^ ^]^ The ions on the left side of the series are well‐hydrated ions and have the ability to precipitate the protein in an aqueous solution, while the right ones (poorly hydrated ions) are able to increase protein solubility. Based on the Hofmeister effect, the significant improvement of mechanical strength and toughness could be achieved by simply soaking the gelatin hydrogel in (NH_4_)_2_SO_4_ solutions (Figure 14e).^[^ ^173^ ^]^ The energy dissipation of the physical crosslinks introduced by the Hofmeister effect is the key for the improvement of mechanical properties, which can pave a new way to fabricate tough and elastic gelatin hydrogels for biodegradable electronics (Figure 14f). Except the physical crosslinks, the introduction of chemical cross‐linkers (e.g., glutaraldehyde, genipin, and tannic acid) also have been demonstrated effective strategies to improve the mechanical properties of gelatin hydrogels.^[^ ^164^, ^166^, ^174^ ^]^

Owing to their high water content and facile processability, gelatin hydrogels with intrinsic ironical conductivity have also been widely used for biodegradable elastic electronics.^[^ ^54^ ^]^ For the practical applications, the considerable mechanical properties, long‐term stability, and environmental tolerance are the important characteristics. The strategies used to improve the mechanical performance of gelatin hydrogels have been discussed before. For long‐term stability and environmental tolerance, the design strategy of organohydrogel is introduced into the preparation of gelatin hydrogels.^[^ ^175^, ^176^ ^]^ Figure 14g showed an ionic conductive gelatin organohydrogel with integrated high‐strength and antifreezing properties.^[^ ^176^ ^]^ The sodium citrate is the key for the preparation of gelatin organohydrogels. It was introduced not only multiple noncovalent cross‐links including physical interactions induced by salting‐out effect and ionic interactions between the −NH^3+^ of gelatin and citrate anions but also endowed the organohydrogel with excellent conductivity. The resultant oragnohydrogels showed a tensile strength of 1.91 MPa, fracture strain of 542%, and ionic conductivity of 0.47 S m^−1^ with sodium citrate concentration of 20 wt%, which would be a promising candidate for the wearable electronics (Figure 14h). The incorporation of the binary solvent of glycerin/water also effectively improved the stability and antifreezing properties with a useful range of temperature even at −30 °C (Figure 14i). The electronic sensor based on this organohydrogel was strain‐sensitive with a large linear sensing window and excellent stability. More importantly, the organohydrogel electronic sensor could be fully recycled due to presence of reversible physical cross‐links.

The chemical modification of grafting methacryloyl onto the gelatin polypeptide chains has been an available strategy to endow the functioned gelatin (GelMA) with photo‐crosslinking properties. GelMA could be easily synthesized through the grafting reaction of methacrylic anhydride and the amine groups on polypeptide chains.^[^ ^177^ ^]^ The GelMA could achieve controllable and rapid gelation by ultraviolet light, which has been one of the most investigated hydrogels for biomedical applications.^[^ ^178^ ^]^ The water solubility and rapid gelation of GelMA make the 3D printing conductive hydrogels available.^[^ ^179^, ^180^, ^181^ ^]^ Shin et al. reported a GelMA/deoxyribonucleic acid (DNA)‐coated CNT hybrid inks with excellent electrical and mechanical properties for various biological and biomedical applications.^[^ ^179^ ^]^ The DNA is used as a natural surfactant to disperse CNTs in GelMA solutions to improve the stability of conductive inks and improve the tolerance to deformation of hydrogels while maintaining decent electrical conductivity. The physical and rheological properties of the conductive ink tuned by adjusting the concentration of GelMA and CNTs to make the 2D and 3D printing of flexible constructs and circuits available.

## 4 Conclusion and Outlook

Biodegradable elastic electronics has attracted significant attention because they are not only proven as a bio‐based solution for e‐waste management and sustainable development but also pave the way for biomedical applications of implantable transient electronic devices. The corresponding biodegradable elastic materials have been recognized as a key to drive this field and will have a wide impact on a range of fields including biomedical engineering, wearable electronics, and soft robots.

Despite the advances of the innovative chemistries that have led a variety of biodegradable elastomers and gels for electronics, the variety and volume of the electric elastic materials with further improved electrical and mechanical properties remains an urgent need, especially the biodegradable conductive and semiconductive elastomers. Synthetic materials possess preferable tunability in overall properties and therefore show the great advantage to construct biodegradable material with desired performance. Although the design strategies of compositing and copolymerization have been demonstrated the availability to fabricate functional elastomers with promising electrical properties, the trade‐off between biodegradability, mechanical properties, and electrical performance is still an urgent problem. Previous works have demonstrated the feasibility of using degradable bonds as linkage to connect the conjugated monomers for fully degradable semiconductors (Type 2 degradation). It is intriguing to choose the suitable degradable and conductive linkage depending on the actual physiological applied environments in future research. The conjugated polymers connected with imine bonds could degrade relatively benign acidic conditions, which showed promising perspectives for the applications of transient electronics. The device using disintegrable polymers based on imine bonds reported by Bao has successfully demonstrated the effectiveness of this strategy for the constructing electronic devices with Type 2 degradation behavior.^[^ ^125^ ^]^ Besides, more conjugated labile bonds, such as vinylene and imidazole bonds that could oxidatively disintegrate exhibit potential for the application in vivo. For example, the biodegradable conjugated polymer nanoparticles for in vivo imaging reported by Kuehne and Pu enabled the feasibility of constructing conjugated polymers with enzymatic degradation.^[^ ^182^, ^183^ ^]^ For the long‐standing competition between the rigid conjugated units and elastic mechanical properties, it is important to consider the methods to achieve the balance of charge mobility and elasticity. Different strategies based on compositing or copolymerizing conjugated polymers/oligomers with biodegradable elastomeric matrix developed by Bao and Lipomi group provided new views for further developments.^[^ ^61^, ^184^ ^]^ Recently, a new molecular design for the elastic semiconductors based on the covalently embedded in situ rubber matrix formation was developed by Bao's group, which enables superior mechanical robustness and electrical performance of elastic semiconductors compared with previous strategies.^[^ ^122^ ^]^

Investigating the relationship between molecular structures and specific properties from the perspective of chemistry to establish the universal principle for materials design would be useful for constructing materials with high electrical performance. Moreover, the adjustment of mechanical properties of the elastic electronic materials is also a major issue for practical applications. Constructing specific biomimetic mechanical performance of electronic devices for different applications would be intriguing for seamless and comfortable integration with human bodies. Endowing biomimetic nonlinear mechanical behavior with improved toughness while keeping the soft nature of elastic electronic materials to mimic the mechanical behaviors of soft tissues have been demonstrated challenging. The brush‐ and comb‐like elastomer developed by Sheiko could precisely encoding the strain‐stress curves to mimic the mechanical behavior of biological materials by adjusting the network strand length, side‐chain length, and grafting density, enabling more mechanical control and synthetic design of synthesized elastomers.^[^ ^185^ ^]^ The in‐depth investigation of synthetic chemistry and the theory of elasticity would be helpful to embrace this challenge. Taking the advantage of first‐principles calculations and artificial intelligence to accurately design materials would be one of the directions of future development in the field of electronic materials. For example, the strategy for the simulation of mechanical properties of cross‐linked networks based on nonequilibrium molecular dynamics simulations reported by Zhang and co‐workers allows accurately simulating and predicting the properties of elastomers before preparation, which would greatly improve the effectiveness of the development of degradable electronic elastomers.^[^ ^186^ ^]^ Besides, it is predictable that establishing specific testing standards and procedures of biodegradable materials would be important for investigating the universal principle between molecular structure and overall performance.

The pursuit and research of new materials would be always the fundamental driving force for the development of this field. Considering the existing biodegradable elastic electronics have used limited materials, such as PGS and natural‐derived hydrogels, it is urging to develop biodegradable electronic elastomeric materials with more functionalities and controllable mechanical and electrical properties by the use of new polymer chemistries. The strategies based on living ring‐opening metathesis polymerization developed by Xia's and Gutekunst's group could construct biodegradable plastic polymers with narrow polydispersities and even distribution of degradable linkages, providing a great opportunity for the preparation of high‐performance biodegradable elastomers.^[^ ^187^, ^188^ ^]^ Moreover, biodegradable polyester elastomers synthesized from lactones reported by Hillmyer and co‐workers exhibited great potential as alternative elastomers for the substrate and dielectric materials.^[^ ^189^ ^]^ The exploration of new catalysts and monomers is vital to apply the precisely controllable polymerization strategies into the biodegradable electronic elastomers. Furthermore, the self‐immolative polymers could disassemble spontaneously through a domino‐like fragmentation based on quinone‐methide elimination induced by extremal stimuli, such as enzymes, chemical analytes, or UV light.^[^ ^190^ ^]^ Developing self‐immolative elastomers and applying these materials into electronics would be an effective solution for the construction of transient elastic electronics with controllable degradation behaviors. Except the biodegradable electronic materials, the recyclable materials also possess great significance for the sustainable development due to their resource conservation. Vitrimer elastomers based on the 3D cross‐linked networks of dynamic covalent bonds exhibited excellent mechanical robustness and thermal reprocessing.^[^ ^191^ ^]^ The dynamic covalent bonds, such as disulfide bonds, transesterification, and Diels–Alder reaction in recyclable vitrimer elastomers enable electronics recycled with stable mechanical and electrical performance. It is also an effective strategy to improve the thermal processability of traditional cross‐linked elastomers, such as PGS, POC by introducing the aforementioned dynamic covalent bonds into their cross‐linked networks.

For the hydrogels, improving the mechanical robustness, environmental tolerance are the aims of research. Very recently, strong tough hydrogels via the synergy of freeze‐casting and salting‐out were reported by He enables the natural‐derived hydrogels comparable mechanical properties of synthesized elastomers, which would be significant for the hydrogel‐based electronics with extremely stable performance.^[^ ^192^ ^]^ In terms of environmental tolerance, the ionic gels have been emerged as important electronic elastic materials and exhibited great potential in the field of wearable electronics. The replacement of water with ionic liquid could avoid the evaporation and freeze of traditional hydrogels significantly improving their environmental tolerance and long‐term stability while maintaining their similar mechanical and electrical properties to hydrogels.^[^ ^193^, ^194^ ^]^ Although their corresponding biodegradability and biocompatibility still need further improvement, it is foreseeable that ionic gels would become promising materials for biodegradable elastic electronics.

With the continuous progress of chemistry and materials, more and more biodegradable elastic materials are expected to be created to greatly promote the development of electronics, biomedicine, and other relevant fields. Though developing new polymer chemistries and expanding the amount of electronic elastic materials are the current problems to be solved, the emerging issues derived from the preparation, usage, and degradation of electronics should also be further considered. For example, the heavy use of toxic solvents for the synthesis of elastomeric materials and the preparation of electronics. Developing solvent‐free or green synthesis of bio‐based or renewable elastomeric materials would be the direction for the next‐generation electronics. Further exploration of biodegradable chemistry would provide the foundation to construct new classes of elastic materials, add further contributions and great opportunities in the era of electronics.

## Conflict of Interest

The authors declare no conflict of interest.
